# A novel approach for proton therapy pencil beam scanning patient specific quality assurance using an integrated detector system and 3D dose reconstruction

**DOI:** 10.3389/fonc.2025.1677439

**Published:** 2025-12-08

**Authors:** Joseph J. Bateman, Sonia Escribano-Rodriguez, Samuel Flynn, Tony Price, Raffaella Radogna, Saad Shaikh, Harry Barnett, Connor Godden, Matthew Warren, Febian, Catherine Burne, Alison Warry, Lee Harrison-Carey, Colin Baker, Simon Jolly

**Affiliations:** 1 Department of Physics and Astronomy, University College London, London, United Kingdom; 2 Radiotherapy and Radiation Dosimetry Group, National Physical Laboratory, Teddington, United Kingdom; 3 School of Physics and Astronomy, University of Birmingham, Birmingham, United Kingdom; 4 Department of Physics, University of Bari, Bari, Italy; 5 Istituto Nazionale di Fisica Nucleare, Sezione di Bari, Bari, Italy; 6 Department of Medical Physics and Biomedical Engineering, University College London, London, United Kingdom; 7 Proton Beam Therapy Physics, University College London Hospital NHS Foundation Trust, London, United Kingdom

**Keywords:** proton therapy, pencil beam scanning, patient-specific quality assurance, integrated detector system, 3D dose reconstruction, Pencil Beam Scanning (PBS) PSQA, scintillator range telescope, CMOS pixel sensor

## Abstract

**Purpose:**

Current proton beam therapy patient-specific quality assurance (PSQA) methods rely on time-intensive phantom measurements or machine-reported parameters without independent verification. This work presents an integrated detector system for phantom-less Pencil Beam Scanning (PBS) PSQA, providing independent spot-by-spot measurements of all critical beam parameters and 3D dose reconstruction.

**Methods:**

The integrated detector combines three separate systems: a scintillator range telescope for range and energy measurement; a CMOS pixel sensor for spot position and size verification; and a Transmission Calorimeter (TC) for beam intensity measurements. Measured parameters feed Monte Carlo simulations to reconstruct 3D dose distributions for comparison with treatment planning predictions. Validation was performed at UCLH using single spot position spread out Bragg Peak (SOBP) and 5×5×10 spot box field configurations.

**Results:**

Energy values obtained from range measurements showed strong correlation with DICOM values (*R*^2^ *>* 0.998) with an accuracy of between 2.17 mm and 1.23 mm for different beam deliveries. CMOS pixel sensor measurements succeeded for single spot fields but experienced saturation at higher intensities and incomplete coverage for the larger box field. The TC demonstrated excellent dose linearity (*R*^2^ = 1.000). Monte Carlo reconstructions agreed well with reference simulations for longitudinal profiles, though lateral reconstructions proved challenging with 77% gamma pass rates (2%/2mm) for the box field.

**Discussion:**

This proof-of-concept demonstrates feasibility of independent beam parameter verification for PBS PSQA while maintaining patient geometry. The approach offers advantages over current methods but requires resolution of energy calibration offsets and detector limitations before clinical implementation. Future work will address these challenges and expand validation to clinical treatment plans.

## 1 Introduction

Despite the theoretical dosimetric advantages of proton beam therapy (PBT) using pencil beam scanning (PBS) (1–3), inherent uncertainties in the proton range — and hence dose conformity around the tumour — limit its full clinical potential (4–6). These uncertainties necessitate conservative safety margins in defining the target volume that can compromise the optimal therapeutic benefits of proton therapy (7–11). To fully leverage the precision capabilities of proton therapy while ensuring patient safety, Quality Assurance (QA) protocols have become increasingly sophisticated, with particular emphasis on patient-specific pre-treatment verification procedures (12, 13). The accurate delivery of PBS fields depends on precise control of multiple beam parameters — including energy, position, spot size, and intensity, all of which are measured individually during regular machine QA — with stringent tolerances required to maintain treatment efficacy (14–16).

The traditional approach to PBS patient-specific QA (PSQA) involves the use of measurement-based pre-treatment verification using either scanned ionisation chambers, or other flat-panel and array detectors, typically within a water or solid water-equivalent phantom (17). Measurement-based PSQA provides a direct dosimetric verification of the delivered treatment beam, and checks the integrity of the entire treatment process from treatment planning to beam delivery (18, 19). However such methods can be extremely time and resource intensive and often fail to pick up errors in the treatment delivery (20, 21). This is largely due to the fact that these measurements are only of point doses or a 2D dose plane rather than the full 3D dose distribution delivered to the patient: comprehensive measurements of the full volumetric dose require multiple redelivery of each field to the instrumented volume. Additionally, such measurements require a modified treatment plan — mostly from the 90° gantry angle — specifically for measurement devices capable of making measurements only from a single direction, as opposed to verifying the actual treatment plan being delivered to the patient geometry (22).

More recently, phantomless log-file-based PSQA methods have enabled a significant reduction in machine QA and PSQA time. Log-file PSQA involves using machine delivery parameters, measured by the beam monitoring system in the machine nozzle either during a dedicated PSQA delivery or during the first treatment fraction (23). These parameters are then used to reconstruct the delivered dose through Monte Carlo (MC) simulations or analytical dose calculation algorithms within the patient geometry from the CT scan (24–28). However, these methods rely on the accuracy of the treatment control system’s self-reported parameters rather than independent measurements, potentially missing certain types of delivery or file transfer errors.

Log-file-based QA — either for machine QA or PSQA — requires data on beam spot position, transverse intensity profile (spot size and intensity distribution) and range/energy for each beam spot. Real-time measurements of spot size and position — alongside delivered dose/Monitor Units (MU) — are normally recorded from the nozzle monitor chambers, with range inferred from the transported beam energy: these are recorded in the log files for each spot during field delivery, with the resulting data used for MC simulation input to reconstruct the volumetric dose. For proper dosimetry, a measurement of absolute dose is also needed to calibrate the relative dose measurement recorded from the monitor chambers. To correctly QA this log-file-based QA, independent measurements should ideally be made of each of these parameters simultaneously: such a QA could be carried out periodically — say as part of weekly QA — with the log-file-based QA then used for daily machine QA and treatment plan PSQA as necessary. The detector system capable of making such a measurement would need to measure pencil beam data quickly enough to resolve individual spots: if separate, independent diagnostic systems are used for measuring different parameters — say, combining a scintillator screen and camera to measure beam spot parameters with a Multi Layer Ionisation Chamber (MLIC) for depth dose — it is essential that each measurement system does not adversely effect the accuracy of the measurement of the others. Such a system would also enable both real-time machine QA and PSQA verification, using the same reconstruction method as used for log-file-based PSQA but with measurements independent of the machine itself. Unfortunately, such a system does not currently exist: detector-based QA measurements of individual parameters are possible, but not at the speed needed for spot-by-spot machine QA and PSQA reconstruction: with single spot dwell times on the order of 5–20 ms for clinical PBS systems, an ideal system would allow sub-millisecond measurements to avoid the risk of blurring between spots.

In this work, a novel integrated detector system — called the Quality Assurance Detector for Proton Beam Therapy (QuADProBe) — along with a novel approach to proton PBS PSQA, designed to provide spot-by-spot measurement of all critical PBS delivery parameters is described. The QuADProBe is designed to measure all the necessary beam parameters for PBT PBS Machine QA simultaneously, as well as being capable of making spot-by-spot measurements for PSQA. The integrated detector system is comprised of three detector components (listed in the order in which they intercept the beam):

1. a Transmission Calorimeter (TC) for measurements of absolute dose (29).
2. a CMOS pixel sensor for spot position and size verification (30).
3. a segmented scintillator range telescope for range and energy measurements (31, 32).

This component order was chosen to ensure that each component was minimally invasive and disruptive to the subsequent components: for example, comprehensive measurement of depth dose is a fully destructive measurement, meaning that by necessity it must be the final component in the detector chain. Minimising the Water Equivalent Thickness (WET) of upstream components alongside the longitudinal gap between diagnostics helps improve the accuracy of the measurement of each component. More detail is given on each of the components in the next section. This enables machine QA to be carried out more quickly and comprehensively, since simultaneous measurements are made of the necessary beam parameters. Part of this speed-up can be achieved by delivering all of the spot positions and ranges to be verified as a single plan; superior validation of log-file based QA can also be achieved through the improved granularity.

For PSQA, the measured beam parameters then serve as input for MC simulations that use individual beam spot measurements for full 3D reconstruction of the delivered dose. This methodology parallels log-file based approaches but with the additional advantage of independently measuring and verifying the delivery parameters, effectively providing a simultaneous QA of the machine log-files, patient treatment plan and beam parameters. The reconstructed dose distribution can then be compared against the treatment planning system’s predicted dose or against a separate MC simulation using the original DICOM plan files as input, providing a robust verification of the entire treatment delivery process.

This approach was experimentally tested at the University College London Hospital (UCLH) Proton Beam Therapy centre (33) using bespoke QA fields: a single spot position spread-out Bragg peak (SOBP) and a uniform dose box field with 10 layers of 5×5 spots with depths from 12–17 cm. These simplified beam delivery plans allowed for straightforward assessment of the detector’s performance, particularly in evaluating the dose reconstruction accuracy in the longitudinal and lateral directions. Similar to phantom-less log-file QA methods (23, 27, 28), by making such a system light enough to be nozzle-mountable, this proposed QA technique would not require the treatment plans to be modified for the pre-treatment verification, reducing clinical resource requirements while maintaining robust quality control.

The following sections detail the integrated detector system design, experimental setup at UCLH, MC simulation methodology, and results comparing the reconstructed dose distributions obtained from simulations using the QuADProBe measured beam parameters with those from the DICOM reference beam parameters. The performance, limitations, and potential clinical implementation of this novel approach to PBS PSQA is then discussed.

## 2 Materials and methods

### 2.1 Integrated detector system overview

**Figure 1:**
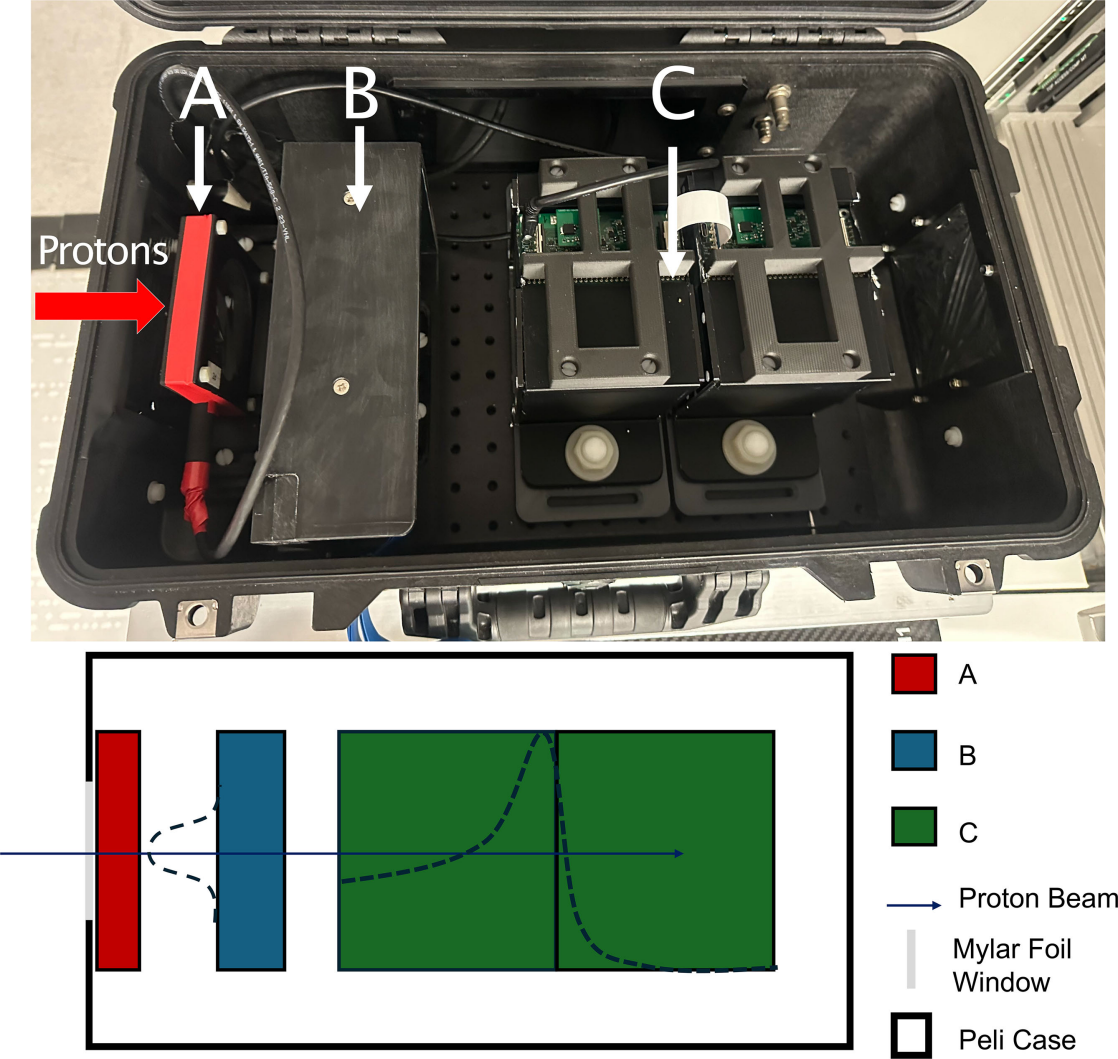
The prototype QuADProBe within a modified Peli case, with aluminised Mylar foil windows. The detector system is comprised of the Transmission Calorimeter **(A)**, the CMOS pixel sensor **(B)** and the QuARC **(C)**. The proton beam enters from the left hand side.

The QuADProBe integrated detector system consists of three complementary detector components housed in a modified Peli 1510 flight case (34), with external dimensions^1^ of 55.9×35.1×22.9 cm^3^. An image of the detector system layout is shown in **Figure 1**. As with the detector design described in (32, 35), a Thorlabs optical breadboard was installed in the base of the case to enable simpler component mounting, and aluminised Mylar foil beam entrance windows installed in the end faces of the case to allow the beam to pass into the detector unimpeded whist retaining the light-tightness of the case. This resulted in a combined detector weight of ~15 kg, with the mass of the case, the optical breadboard and the actual detector components each representing approximately one-third of the total mass. While the external structural features of the case restricted the size of the beam entrance windows to 10×10 cm^2^, modifying an off-the-shelf flight case to accommodate the experimental setup proved to be a more effective way of realising a portable detector than designing a custom enclosure.

Care was taken in mounting the optical breadboard to ensure equal distance between the base of the case and the mounting surface of the breadboard along the length of the case meaning that any flat surface upon which the enclosure was placed — such as the treatment couch — would be parallel with the detector components. Beyond the steel bolts used to hold the metal optical breadboard in place, nylon bolts were used throughout to minimise beam scatter from beam falling outside the boundary of the entrance windows. Alignment markings were also added to the exterior of the case to ensure accurate alignment with in-room laser systems and repeatability of system setup.

The components are arranged sequentially along the beam path: first the Transmission Calorimeter (TC) positioned directly behind the Mylar foil entrance window, followed by the CMOS pixel sensor (LASSENA), and finally the Quality Assurance Range Calorimeter (QuARC) scintillator range telescope at the rear of the setup. This configuration enables simultaneous measurement of beam spot size, position, intensity and energy for each delivered pencil beam spot.

The QuADProBe system provides near real-time measurement capabilities with varying temporal resolution across detector components. The QuARC detector operates with a 170 µs integration time, enabling 6 kHz real-time energy measurements for individual energy layers during delivery, with calibrated depth dose data displayed on a live GUI at 25 Hz refresh rate. The CMOS pixel sensor operates at readout speeds of 35 fps (full frame) to 230 fps (ROI mode), while the TC acquires temperature data at 10 Hz. Data acquisition occurs during beam delivery (typically 30 s–3 minutes for the fields tested), with post-processing analysis required for both CMOS spot parameter extraction and TC dose measurements.

### 2.2 Transmission calorimeter

Calorimeters for proton therapy — by which absolute dose deposition is measured through small changes in temperature in a controlled medium — have been under development at NPL for a number of years (36, 37). However, while the primary standard graphite calorimeter provides the basis for the NPL dosimetry calibration service under the IPEM code of practice (38), the size and complexity of the device makes it unsuitable for daily use. As such, smaller devices have been under development at NPL to supplement the primary standard dosimetry measurement (39, 40).

The Transmission Calorimeter (TC) measures absolute dose through radiation-induced temperature changes, and operates on the principle that the energy absorbed by the calorimeter core is less than or equal to the energy that would be absorbed by transmitting the radiation through 2 mm WET (29). This enables minimal beam perturbation for the remaining detector components while maintaining measurement sensitivity.

**Figure 2:**
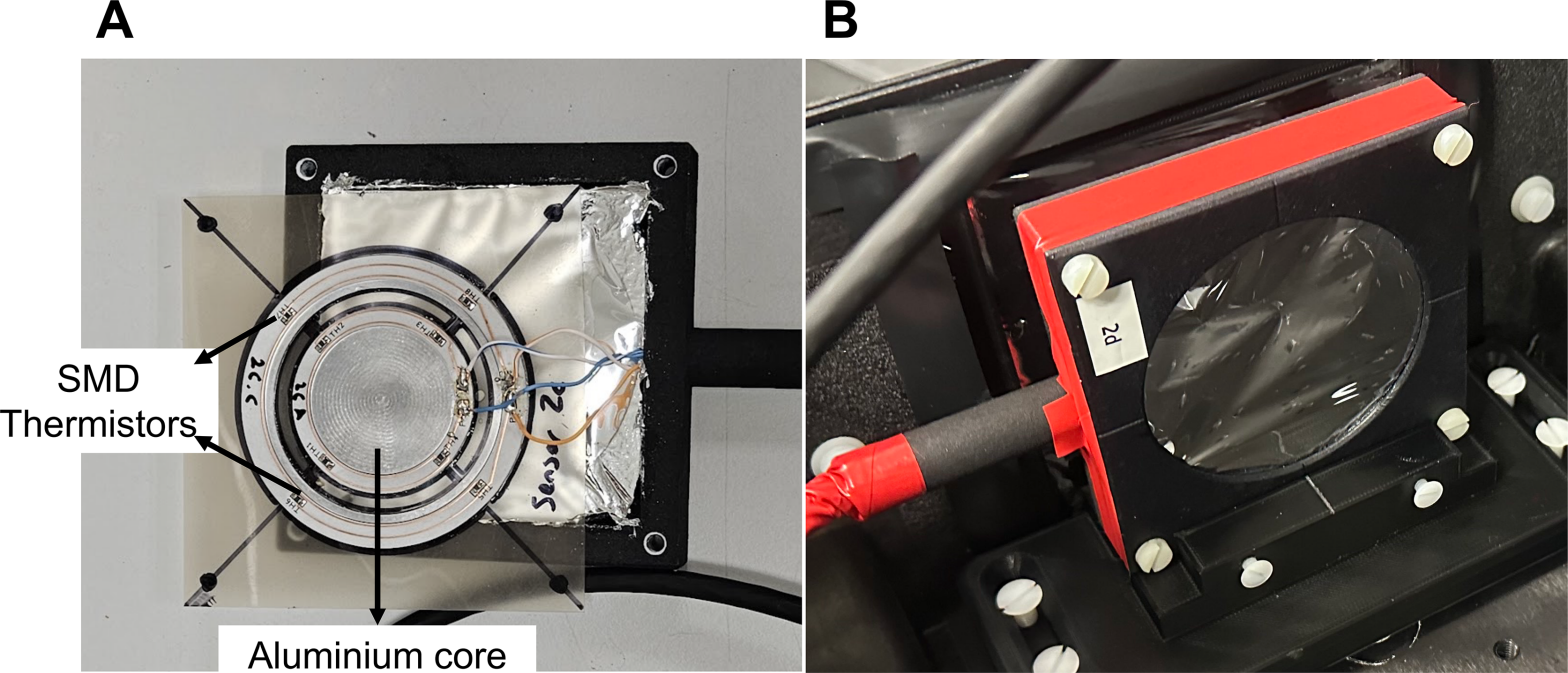
**(A)** Photograph of the sensitive components of the transmission calorimeter showing the aluminium calorimeter core in the centre and the surrounding periphery whereby the SMD thermistors are mounted, two of which are labelled and the other two are on the opposite side. **(B)** Photograph of the transmission calorimeter within its casing with an aluminised Mylar foil window.

The calorimeter core, shown in **Figure 2A** consists of a 45 mm diameter aluminium printed circuit board (PCB), which has mass stopping power of 7.41 MeV·cm^2^/g for protons and a WET of approximately 1 mm. Four SMD thermistors (10 kΩ at 25°C) are mounted at the periphery of the PCB to measure the radiation-induced temperature change of the core. The thermistors are networked together in a bridge configuration to measure average resistance change, without readout performed using a DC bridge excitation voltage of +10V. The excitation voltage was measured using a Keithley 6500 digital multimeter, configured with an acquisition frequency of 10 Hz. This allows for sensitivity to small temperature variations while maintaining measurement stability.

The system incorporates active temperature compensation to account for ambient temperature fluctuations during measurement. The bridge-out-of-balance voltage was calibrated in an environmental chamber traceable to the NPL primary standard for temperature across the operational range of 18–30°C. The calorimeter measures dose-area-product, with the measured temperature change (Δ*T*) being directly proportional to the deposited dose according to:


$$\text{D}\propto \frac{\text{Δ}T\times C_{p}}{\rho \times A}$$

where *C_p_* is the specific heat capacity of aluminium (0.900J/g·°C), *ρ* is the density, and *A* is the effective sensing area, but only applies when A is smaller than the beam size.

The TC assembly, shown in **Figure 2B**, was mounted just inside the entrance window within the Peli case. This was then connected through a patch panel port on the side of the Peli case to the power supply and a pair of high precision voltmeters via a single multi core cable to allow the larger measurement devices to be mounted suitably far from the irradiated detector volume.

Given the small temperature changes associated with the energy deposited in the thin calorimeter core, measurement times on the order of seconds are needed. This is clearly much longer than the sub-millisecond timescales needed for spot-by-spot measurements mentioned in the Introduction. The intention was therefore to make a long measurement with all the detector components with a single spot and then calibrate the integrated intensity measured by both the pixel sensor and QuARC to the absolute dose measured by the TC. This calibration could then be used to scale the relative intensities measured by the two “real-time” detectors to provide absolute rather than relative dose.

### 2.3 CMOS pixel sensor

**Figure 3:**
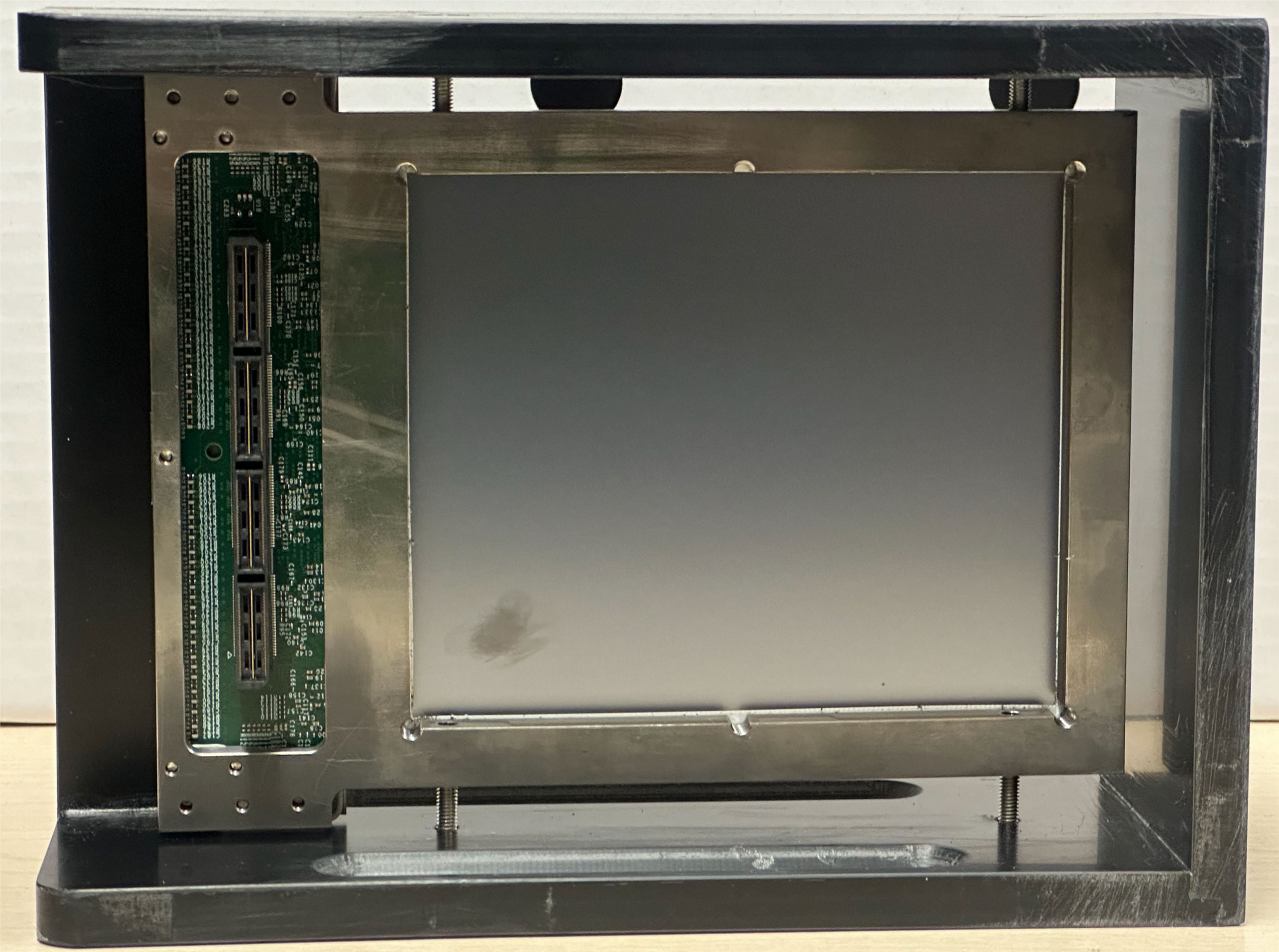
Photograph of the rear of the large-format vM2428 CMOS pixel sensor from the beam’s perspective.

The position and size of each delivered proton beam spot was measured using LASSENA, a large-format vM2428 Complementary Metal-Oxide-Semiconductor (CMOS) pixel sensor (30), shown in **Figure 3**. The CMOS sensor has a 50µm pixel pitch with an active area of 120×140 mm^2^ (2400×2800 pixels), a WET of ~1.5 mm, and a full frame readout speed of 35 fps, which can be increased up to 230 fps when using a limited region of interest (ROI) of 120 mm wide and 20 mm tall. The readout board is external to the pixel sensor itself, with data acquisition provided through a frame grabber PCI card connected to the readout board: this was mounted outside the Peli case with the cabling between the pixel sensor and readout board routed through a light-tight aperture in the side of the case.

This pixel sensor was selected since its active area was larger than that of the QuARC (see below) and because an existing DAQ chain was already well-established at the University of Birmingham, as well as being previously tested at the UCLH PBT centre (41). The pixel sensor and associated readout could also be integrated relatively easily with the QuARC setup. For each delivered spot, the CMOS detector provides a 2D intensity distribution, to which a Gaussian fit is applied in both X and Y and the proton pencil beam spot size and position obtained from the respective mean and standard deviation of the distribution. A full 2D detector has the advantage over a pair of 1D measurements (as is normally implemented in a clinical nozzle) that non-uniformities that would otherwise be hidden by independent X and Y measurements — such as hollow beams — are revealed with a true 2D measurement; it also halves the number of components that could disrupt the beam measured by downstream components. A pixel sensor has the advantage over a scintillator screen that a scintillator screen either needs a camera placed directly in the beamline or the introduction of a mirror to allow off-axis measurement: both would increase both the longitudinal size of the detector and the number of components that can cause proton beam scattering and energy straggling. The disadvantage is that there is a quadratic growth in data points over an orthogonal pair of 1D arrays with the same resolution (2400 × 2800 = 6.72 million pixels compared to 5,200 rows + columns), placing more stringent requirements on the readout system. Additionally, the architecture of the CMOS is based on a 3T pixel design and is sensitive to rolling shutter artefacts due rows being read asynchronously.

Previous investigations into its viability for proton therapy beam monitoring at UCLH (41), and for proton minibeam radiotherapy (pMBRT) at Institut Curie (42), demonstrated the detector’s ability to provide high spatial resolution measurements of proton beams as well as provide beam size measurements that agree to better than 0.25 mm with those made with EBT3 radiochromic films. For single spot position SOBP field measurements, the detector was operated using its maximum readout speed using the 120 mm×20 mm ROI. However, for scanned fields, the ROI was too small, so the full frame readout using the slower readout speed of 35 fps was used for these measurements. This relatively slow frame rate when compared to both the scanning speed of a single treatment plan energy layer and the readout speed of the QuARC (see below) prevented individual spots being measured in real time. Instead, for scanned beams with dwell times shorter than ~30 ms individual spot profiles had to be extracted from frames that integrated more than one spot during scanning.

### 2.4 Scintillator-based range telescope

**Figure 4:**
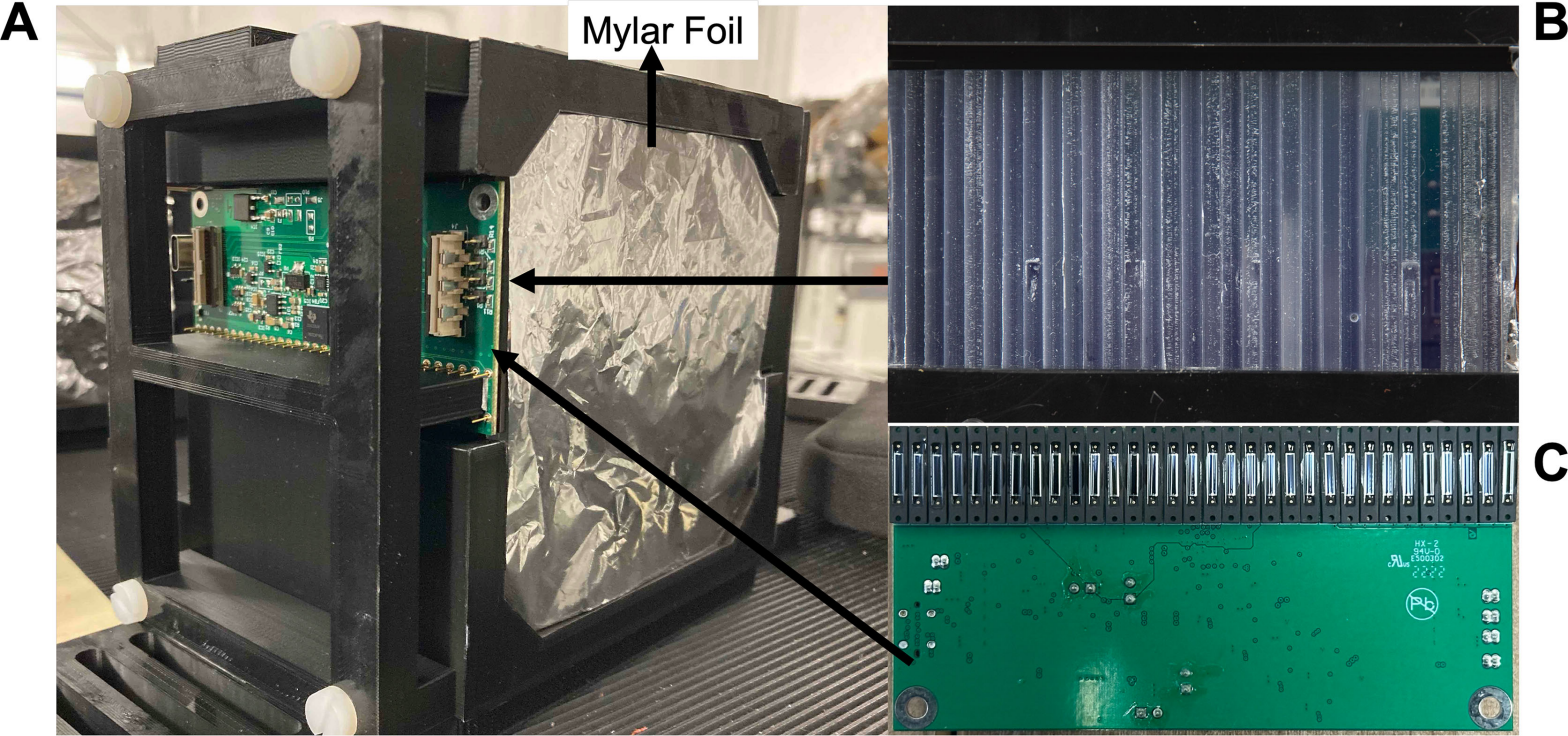
**(A)** Single QuARC detector module within the 3D-printed casing with the aluminised Mylar foil covering the front and photodiode board mounted to the side. **(B)** Side view of the scintillator sheets stacked within the module (with aluminised Mylar foil inserted between each sheet). **(C)** Photodiodes mounted to DDC232 front end ADC board.

The QuARC is a scintillator-based range telescope comprised of a stack of scintillating sheets, designed for fast water-equivalent proton range measurements with sub-millimetre accuracy (31, 32). For this experiment, a QuARC consisting of two modules with 32 NuDET SP32 scintillator sheets (43) — each 100×100×3 mm^3^ — was used, shown in **Figure 4**.

The combined water-equivalent thickness of the two modules was approximately 197.4 mm (using the measured relative stopping power of protons to water of 1.024). Each scintillator sheet is optically isolated using 6 µm thick aluminised Mylar foil (see **Figure 4C**) and housed in a custom 3D-printed black PLA holder. The scintillation light is detected through the use of Hamamatsu S12915-16R photodiodes (44) (see **Figure 4C**), that are coupled directly to each scintillator sheet; photodiode current integration is then performed using Texas Instruments DDC232-CK current-integrating analogue-to-digital converters (45), providing 20-bit precision with integration times between 166.5 µs and 1s. The DDC232 circuit boards are then read out using a Nexys Video FPGA development board (46), with data acquisition (DAQ) performed on a Raspberry Pi5 (47), allowing photodiode charge to be acquired at rates of up to 6 kHz. A web-based graphical user interface (GUI) provides a live display of the calibrated photodiode charge levels and real-time proton range reconstruction using live fitting at 25 Hz.

The energy of each of the proton pencil beams was determined through the analysis of depth-light curves produced by the QuARC photodiodes. After background subtraction and calibration of the photodiode outputs (see (32)), each depth-light distribution was processed to determine the beam energy. The energy determination process uses the numerical model developed by Kelleter and Jolly (48) — called the “quenched Bragg” (QB) model — which takes Bortfeld’s mathematical approximation of the proton Bragg curve (49) and applies Birks’ law for scintillation light quenching within the scintillator sheets. The function is then fitted to the measured photodiode charge values, with each data point representing the integrated scintillation light from a single ~3 mm thick scintillator sheet. Rather than treating each measurement as a point value at a specific depth, the fitting algorithm accounts for the fact that each photodiode measurement represents the integrated dose deposition across the entire thickness of its corresponding scintillator sheet. This approach compensates for the detector’s finite longitudinal spatial resolution of approximately 3 mm per measurement bin. The model accounts for the non-linear scintillation light output in regions of high linear energy transfer (LET) around the Bragg peak.

Using Bortfeld’s established range-energy relationship for therapeutic protons energies (49), the fitted range value (*R*_0_) was converted to the corresponding beam energy, using:


$$R_{0}=\alpha E^{p}$$

where E is the energy of the proton beam, $\alpha \;\left(=1.742\text{ cm }{\text{MeV}}^{-p}\right)$ is the proportionality factor and p (=0.02543) is the exponent of range-energy relation. This provided an independent verification of the delivered beam energy for each field, with an estimated uncertainty of ± 0.2 mm in range determination, translating to approximately ± 0.3 MeV energy uncertainty at the proton energies used in this study.

### 2.5 Experimental setup at UCLH Proton Therapy Centre

**Figure 5:**
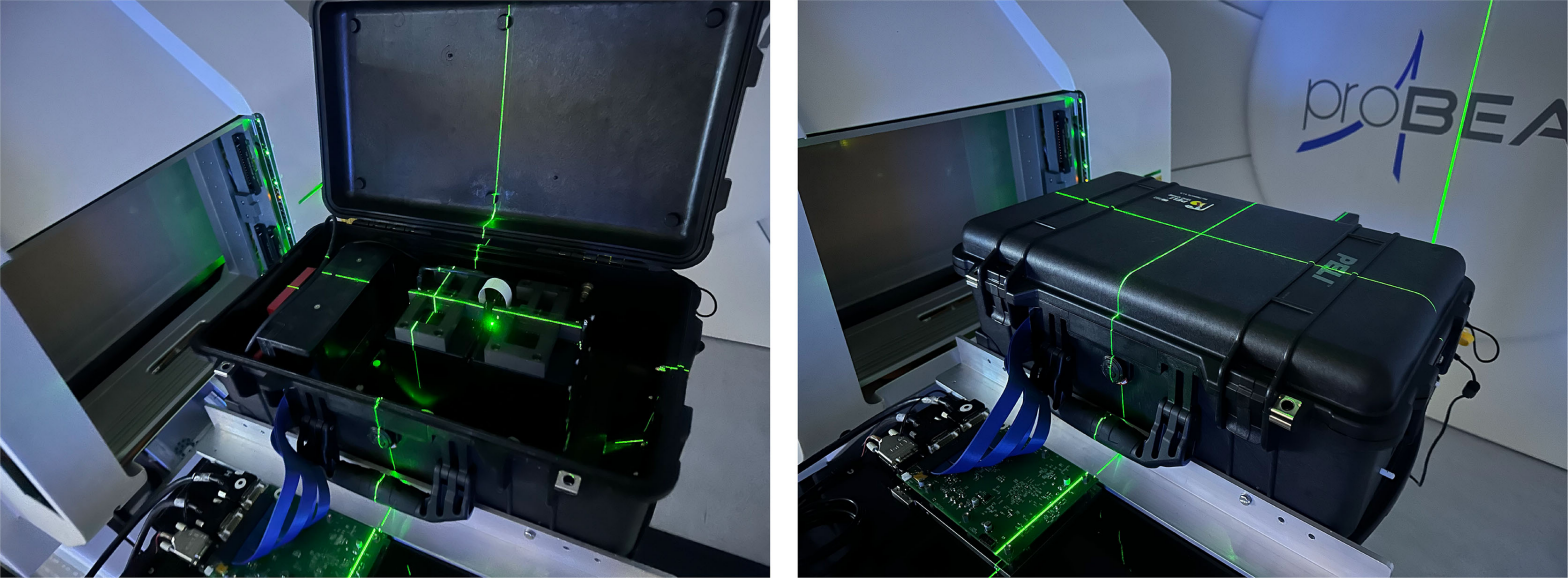
QuADProBe detector situated in front of the UCLH Varian ProBeam Gantry with the Peli case closed (left) and open (right). The case was positioned such that isocentre sat in the centre of the first QuARC module inside the case. The pixel sensor readout board sits in front of the QuADProBe case in both images.

Measurements were performed over 2 consecutive evenings in gantry treatment rooms 1 and 3 at the UCLH proton beam therapy centre. The detector, enclosed within the Peli case, was positioned on the treatment couch and was aligned such that the isocentre was positioned in the centre of the first QuARC module, as seen in **Figure 5**. The couch was positioned to ( −11.80 cm, +96.45 cm, 0.00 cm, 0°, 0°, 0°), and the gantry was rotated to 270° — the 9’o’clock position as viewed from the treatment room toward the gantry — to ensure that the direction of the proton beam was perpendicular to the QuADProBe entrance window.

The main beam configurations used were a single spot position SOBP with 34 energies in the range 72.04–159.55 MeV, and a box field with 10 energy layers ranging from 126.26 MeV to 152.78 MeV and with lateral spot positions from −2 to +2 cm with 1cm spacing in both the horizontal and vertical directions. An additional box field with 7 energy layers ranging between 134.98 MeV and 152.78 MeV with lateral spot positions from −1 to +1 cm with 1 cm spacing was delivered for the purpose of investigating the response linearity of the TC. All of these treatment plans are shown in **Supplementary Figure S1**.

Several issues were experienced during the two evenings. While the same room was scheduled for both evenings, a persistent couch position interlock on gantry 1 during the second evening of measurements rendered gantry treatment room 1 unusable. This meant that while validation of individual detector components could be carried out during the first evening on gantry 1, for the full system measurements — that form the bulk of the combined measurements described below — the setup had to be moved to gantry 3 at extremely short notice, with no time available to repeat the planning for the alternative gantry. This meant a slight discrepancy between the spot sizes in the DICOM plans and the measured beams for the box fields, affecting the subsequent volumetric dose reconstruction.

In addition, substantial time was lost on the first evening resolving electronic noise pickup issues with the QuARC, meaning that combined measurements of the full detector were not possible until the second evening (this was only resolved with a subsequent redesign of the QuARC front-end electronics). This in turn prevented measurements being repeated during the second evening since there was only a fixed 4 hour window for measurements after the end of the treatment day and because beam was being shared with QA measurements in other treatment rooms. Finally, an automatic triggering system to synchronise acquisition between the pixel sensor and the QuARC — and the initiate acquisition suitably far in advance of the arrival of the beam — was not available, meaning that acquisition for each component had to be triggered manually. This in turn meant that, on occasion, the very start of an acquisition was missed by one of the components: this resulted in a missed energy layer by the QuARC in the single spot position SOBP field and issues with the CMOS sensor in box field reconstruction that was only picked up during post-measurement data analysis.

### 2.6 Monte Carlo simulations and comparative metrics

MC simulations were performed with OpenTOPAS (v4.0.0) (50, 51), using the default physics list, which have been optimised and extensively validated for proton beam therapy PBS dose simulations (52, 53). To reconstruct the 3D dose distribution, the measured beam parameters for each proton beam spot within each delivered field served as the primary input to the MC simulations: namely the beam size and position obtained from the beam profile measurements using the CMOS pixel sensor, and the beam energy and energy spread from the range measurements using the QuARC. These results were then compared to 3D dose distributions reconstructed from MC simulations using the DICOM-specified beam parameters. 10^6^ simulated protons were used per proton beam spot.

The relative intensity of the individual spots were scaled using the MU weights from the DICOM file, additionally for the single spot position SOBP field the spots were also scaled by the spot intensity as measured by the CMOS pixel sensor and the QuARC detector. The resulting distributions were then normalised for the purpose of the analysis in this investigation. The beam source position was taken to be that of the CMOS pixel detector, therefore being defined as 15cm upstream from the isocentre. Since the DICOM beam sizes are given for that at isocentre, using the virtual point source distance, the DICOM input beam spot sizes were back-projected to the position of the CMOS detector (the position of which was used as the source for the QuADProBe MC simulations) using the virtual source distance from the DICOM file. The dose was scored within in a 20×20×20 cm^3^ water phantom, with front face of the water phantom positioned at isocentre. The dose was scored in bins of 1 mm^3^. The MC reconstructed 3D dose distributions were then normalised to the respective maximum dose value and compared as relative dose distributions rather than absolute dose. This approach was adopted because:

1. the primary objective was to validate the geometric accuracy of dose reconstruction using independently measured beam parameters rather than absolute dosimetry.
2. the current timing resolution of the TC readout system does not permit individual spot-by-spot absolute dose measurements during rapid PBS delivery sequences.
3. relative dose comparisons allow direct assessment of the spatial accuracy of the reconstructed dose distributions, which is the critical parameter for patient-specific quality assurance applications.

Monte Carlo dose reconstruction was performed using OpenTOPAS with 10^6^ protons simulated per pencil beam spot. Each individual spot simulation required 1–4 hours of computational time depending on beam energy and was submitted as an independent job to the UCL High Energy Physics batch farm, which consists of 23 DELL PowerEdge servers (Intel Xeon processors, 48–64 GB RAM). This parallel job submission enabled simultaneous processing of all spots within a field, with total wall-clock time for complete field reconstruction limited primarily by the longest-running individual spot simulation rather than cumulative computational time. In order to compare the reconstructed dose distributions from the QuADProBe and DICOM inputs, key parameters as described in the AAPM TG-224 (14) are used. For the longitudinal dose profiles, the beam range in water is defined as the depth at 80% distal dose and the distal dose falloff width is the distance along the longitudinal axis whereby the dose drops from 80% to 20%. Additionally for the single spot position SOBP, the width of the SOBP is calculated as the longitudinal distance in water between the 90% dose at the proximal and distal edge of the SOBP. For the lateral profiles, the single spot position beam sizes were obtained by applying a 1D Gaussian fit to the profiles and calculating the mean and standard deviation. For the box field, the size was determined as the full-width at half maximum (FWHM), along with additional calculations of the lateral penumbra width where the lateral dose reduces from 80% to 20%. Additionally, the lateral uniformity of the box field was calculated across the central 80% of the box field beam filed size (defined as the FWHM), using the lateral flatness, *F_lp_*, defined as:


$$F_{lp}=\left(\frac{d_{lp,max}-d_{lp,min}}{d_{lp,max}+d_{lp,min}}\right)$$

where $d_{lp,max}$ and $d_{lp,min}$ are the maximum and minimum dose values in the lateral beam profile; and the lateral symmetry, $S_{lp}$, defined as:


$$S_{lp}=100\times \left(\frac{D_{1}-D_{2}}{D_{1}+D_{2}}\right)$$

where *D*_1_ and *D*_2_ are the integrated doses in each half of the lateral profile about the central axis. Lastly, a gamma evaluation was performed to compare the shape and uniformity of the box field profile from the QuADProBe dose distribution reconstruction (the evaluation plan) with that of the DICOM reconstruction (the reference plan) (54). A 2%/2mm criteria for the gamma analysis was set, according to recommendations in AAPM-TG224 (14), with a 5% dose cut off threshold. The gamma analysis was performed using the PyMedPhys library (55).

## 3 Results

### 3.1 Absolute dose

**Figure 6:**
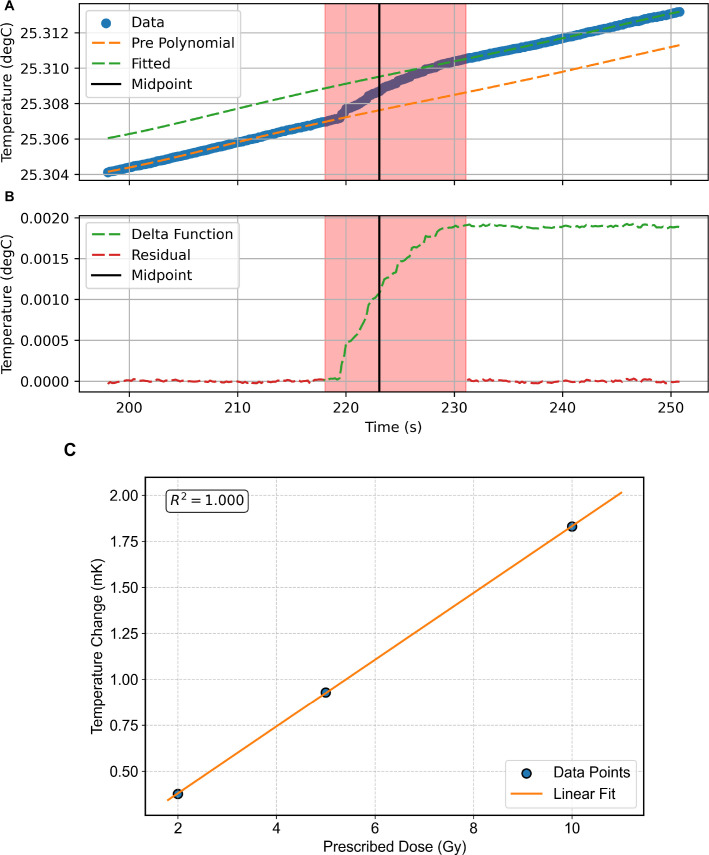
Transmission calorimeter temperature **(A)**, with polynomial fits applied to the pre- and postirradiation thermistor drift and temperature change, and **(B)** as a function of time, with the residuals for these fits shown, whereby the red shaded area denotes the beam irradiation time. **(C)** shows the temperature change and a function of the prescribed dose in a 3×3 spot cube field.

The TC successfully demonstrated dose-area-product measurement through radiation-induced temperature changes during proton beam delivery. **Figure 6A** shows the temperature profile recorded during beam delivery. The pre-polynomial fit is applied to the pre-irradiation temperature drift of the thermistor, and another polynomial fit is applied to the post-irradiation temperature drift. The temperature change is then determined as the difference between these two fits. **Figure 6B** shows the delta function (i.e. temperature change while the beam is on) along with the residuals of the pre-polynomial fit applied to the temperature drift prior to irradiation, and the fit applied to the post-irradiation temperature drift. **Figure 6C** shows the correlation between measured temperature change and prescribed dose within a 3×3×3 cm^3^ volume, showing a linear relationship (*R*^2^ = 1.000). This validated the TC’s quantitative dose measurement capability for intensity scaling in Monte Carlo dose reconstruction. The linear response demonstrates suitability for real-time dose monitoring across clinical beam intensities in proton therapy PBS PSQA.

These measurements were performed in order to assess and quantify the accuracy and linearity of the dose measurement capability. However, these measurements were not incorporated into the Monte Carlo dose reconstruction workflow due to the timing resolution of the readout system, which does not allow individual spot-by-spot dose measurements during the rapid beam delivery sequences used in pencil beam scanning. Given the large time resolution differences between the TC and the other detector components, the intention for future measurements is to calibrate the relative intensities from both the profile monitor and range telescopes to a single absolute dose measurement from the TC, then apply a suitable scale factor for the spot-by-spot measurements to provide an absolute dose measurement for all spots.

### 3.2 Beam spot size and position

**Figure 7:**
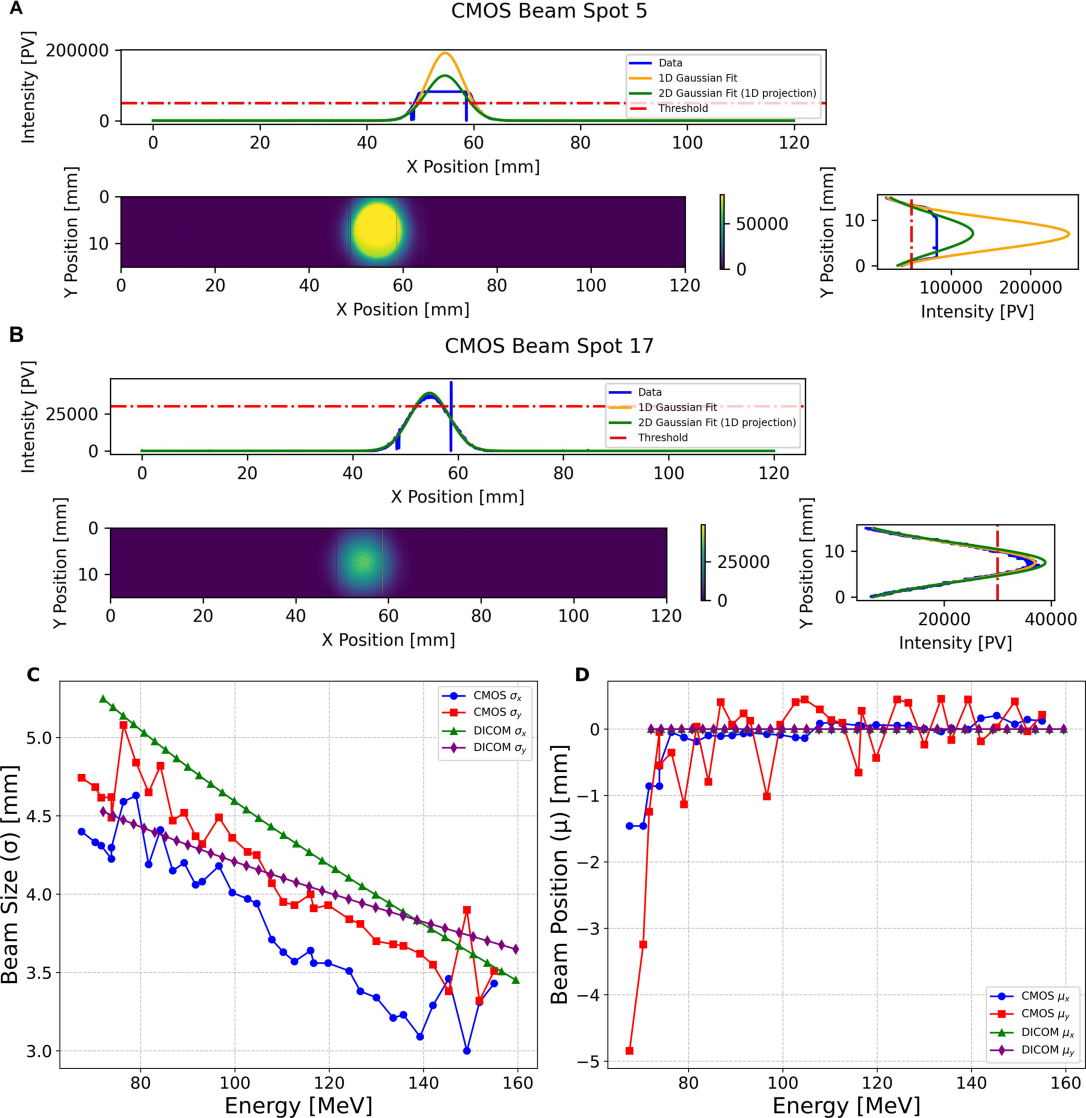
CMOS pixel sensor 2D profile measurement of **(A)** a 147.5MeV and **(B)** 113.0MeV pencil beam spot, showing the 1D profiles in the X and Y direction, and applying the Gaussian fits for a 1D slice and 2D projection. **(C)** shows the pencil beam spot size and **(D)** position measurements of the proton pencil beam spot size and positions for the 34 pencil beams in the single spot position SOBP plan, obtained from applying a Gaussian fit to a 1D slice across the centre of the beam profile in X and Y.

Measurements of the spot size and position for the single spot position SOBP field using the CMOS pixel sensor were performed using the 120 mm×20 mm ROI, meaning the detector could be operated at the maximum readout speed of 230 fps. The beam profile was averaged over the number of frames that accounted for the duration of each beam spot (corresponding to a single energy layer in the SOBP plan); during post-processing a Gaussian fit was then applied to each profile, from which the size (*σ*) and position (*µ*) are obtained. As shown in **Figure 7**, two types of Gaussian fit were applied in each the X- and Y-direction: the first being applied to a 1D slice in each direction centred on the mean, and another being the projection of a 2D Gaussian fit. For the purpose of this experiment only the 1D slice fits were used as these were least impacted by the detector saturation effects.

Due to greater beam intensity for the higher energies in the SOBP field, the CMOS detector experienced saturation for some of these measurements (see **Figure 7A**); however, it was still possible to apply a Gaussian fit to these profiles to obtain the beam size and position. **Figure 7B** shows the unsaturated beam spot profile for one of the lower energy spots in the delivered field with an energy of 113.01 MeV. **Figure 7C** shows the comparison of the CMOS sensor measured beam sizes for the single spot position SOBP field, compared to those in the treatment plan DICOM (back-projected from the isocentre to the position of the CMOS sensor using the virtual source distance). With the exception of one measurement for *σ_x_* and three for *σ_y_*, the beam size measured by the CMOS is smaller than that described in the DICOM. **Figure 7D** shows the comparison of the beam positions measured by the CMOS sensor for each spot for the same field: in this instance the CMOS measured positions fluctuate significantly — especially *µ_y_* — despite all of the prescribed DICOM positions being at (0,0) mm.

The CMOS pixel sensor measurements of the spot size and position for the 5×5 spot box field were performed using the full frame readout of 120 mm by 140 mm due to the larger area of the field: this meant however that the maximum readout speed was limited to 35 fps. Furthermore, since this box field included lateral scanning of the pencil beam, this meant that a significant number of the spots were either distorted (as seen in **Supplementary Figure S2**), unable to be deciphered, or completely missed by the CMOS pixel sensor. Unfortunately, limitations in the flexibility of the planning software and lower limits on beam monitoring within the nozzle prevented slower scanning speed with reduced current: the only available method of increasing the scanning time for each energy layer was to vastly increase the total dose for the whole plan, which would have resulted in far more layers saturating the CMOS sensor. As such, instead of measuring 25 spots for 10 energy layers, only 16 spots in the highest energy layer were deemed suitable for evaluation. Subsequently for the purpose of the Monte Carlo Dose Reconstruction, a subset of the correctly measured spot consisting of a single layer of 3×3 spots out of the 16 usable spots were chosen for the lateral profile analysis for broad fields.

### 3.3 Range and energy

**Figure 8:**
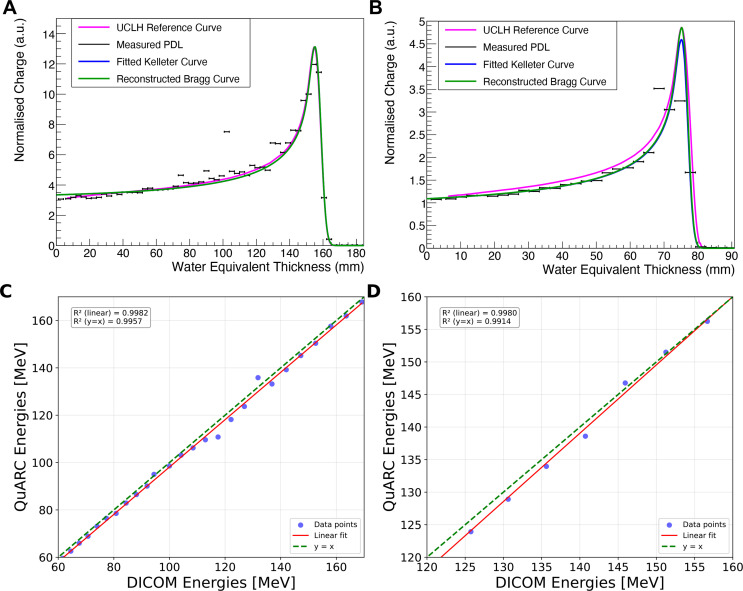
Plots showing the QuARC energy measurements for two of the pencil beams in the single spot position SOBP, **(A)** with a QuARC measured range of 76.46 mm and corresponding energy of 99.22 MeV compared with a reference energy Bragg curve for 100 MeV **(B)** with a QuARC measured range of 157.58 mm and corresponding energy of 150.28 MeV compared with a reference energy Bragg Curve of 150 MeV. The blue curve represents the fitted QB function, and the green curve is the reconstructed Bragg curve using the Bortfeld fit, and the purple curve is the reference energy Bragg curves from the UCLH PBT facility. **(C)** shows the correlation between the proton beam energy measured with the QuARC detector, and the reference energy reported in the DICOM file, for the single spot SOBP field, and **(D)** the 5×5 box field. Each has a linear fit applied and *R*^2^-value calculated relative to the linear fit, and the unity line, that is shown in **Table 1**.

For the single spot position SOBP field the QuARC detector measured the average percentage depth light (PDL) curve over the duration of each of the 34 pencil beam spots (corresponding to each of the different energies). For the 5×5 spot box field, the QuARC detector measured the average PDL curve for each of the 10 energy layers, meaning that a single average energy was used when reconstructing the volumetric dose of the 25 spot lateral positions in each energy layer. **Figure 8** shows the QuARC energy measurements from the single spot position SOBP field with energies of 99.2 MeV (A) and 150.3 MeV~(B).

The prescribed DICOM pencil beam spot energies against the corresponding energies measured by the QuARC detector for the single spot position SOBP field are shown in **Figure 8C**, with the equivalent for the 5×5 spot box field show in **Figure 8D**. The corresponding parameters for both the linear fits are shown in **Table 1**. Both linear fits have a gradient close to unity, indicating that the WET of the QuARC is extremely well characterised. The resulting intercept for these linear fits is −2.372 MeV for the SOBP field and −7.884 MeV for the box field, corresponding to a water equivalent range offset of -1.89 mm and -7.83 mm, respectively. The larger offset seen for the 5×5 box field is a result of the substantially higher minimum energy needed for the proximal energy layer of the box field (126 MeV) compared to the lowest energy used for the SOBP (72 MeV) which, combined with the lower number of datapoints, makes the linear fit more sensitive to small changes in reconstructed range.

**Table 1:** Parameters of the linear fit applied to the correlation between the prescribed DICOM energies and the QuARC measured energies for the single spot position SOBP field and 5×5 spot box field in **Figures 8C, D**.

Linear fit parameter	Single spot pos. SOBP	5×5 spot box field
Slope (a.u.)	1.009	1.052
Intercept (MeV)	-2.372	-7.884
*R*^2^-value (linear fit)	0.998463	0.998037
*R*^2^-value (unity)	0.995609	0.990900

**Figure 9:**
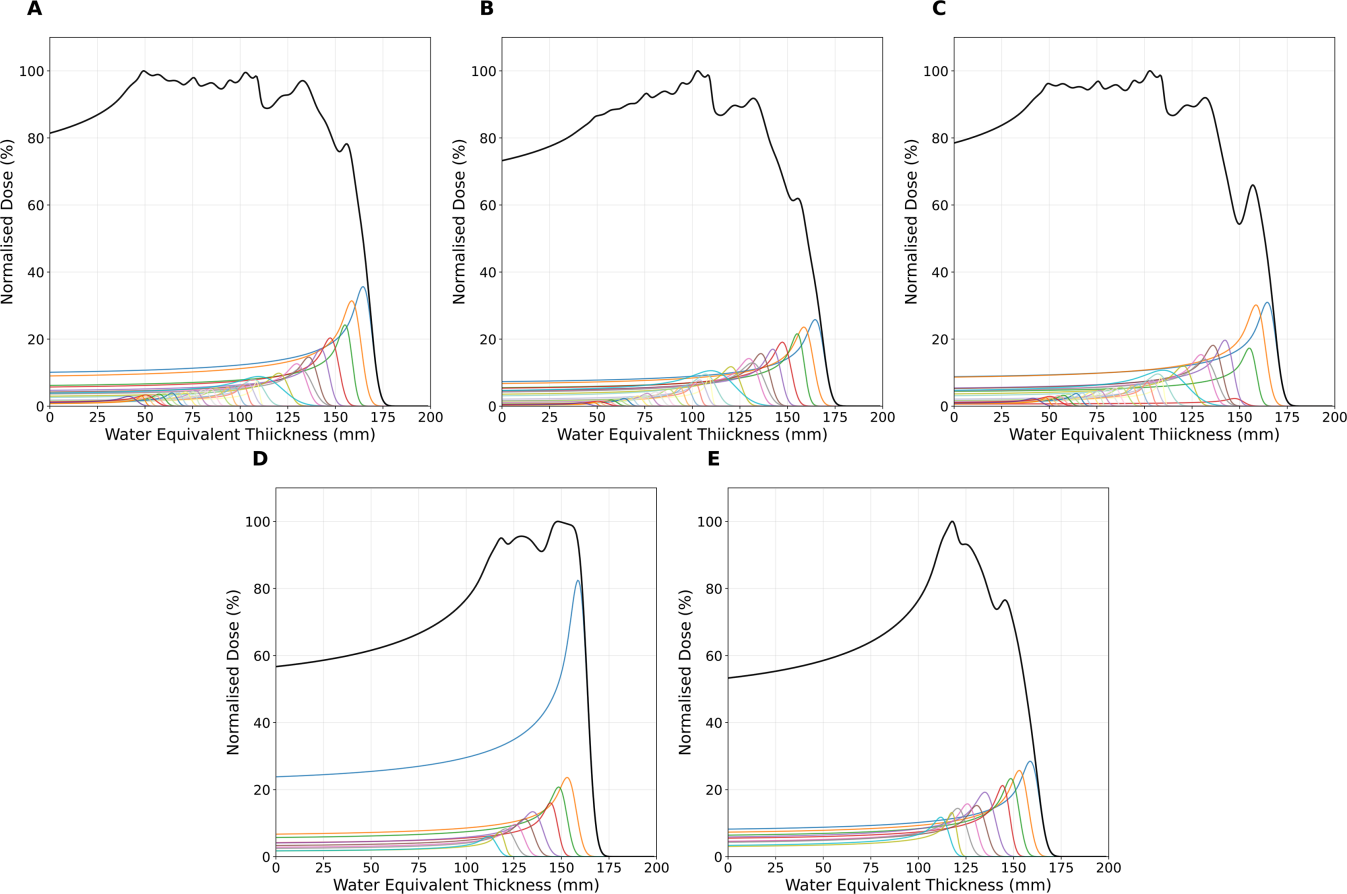
Reconstructed SOBPs (black lines) from QuARC energy measurements showing individual Bragg curves (coloured lines) for each energy layer. **(A–C)** show the single spot position SOBP field with Bragg curves weighted by: A DICOM prescribed MU weights, B integrated CMOS sensor intensity, and C integrated QuARC intensity. **(D, E)** show the 5 × 5 spot box field weighted by D DICOM MU weights and E QuARC integrated intensity. Note the missing highest energy Bragg peak in subfigures **(A–C)** due to QuARC acquisition failure during the SOBP delivery.

To assess the impact of energy measurement accuracy on dose reconstruction, SOBP profiles were generated from the individual Bragg peak measurements. **Figure 9** shows the reconstructed SOBPs and their constituent Bragg curves for different intensity weighting schemes. It should be noted that for the single spot position SOBP field, the QuARC detector failed to acquire data for the first (highest energy, most intense) energy layer due to the manual triggering synchronisation issues described in Section 2.5. This can be seen in **Supplementary Figure S3**, which shows the acquired energy layers and only 33 energy layers are present for the SOBP field as opposed to the delivered 34. The absence of the 159.55 MeV Bragg curve creates a pronounced dose deficit in the proximal SOBP region across all weighting methods (**Figures 9A–C**), which compromises the dose uniformity along the SOBP plateau. The comparison of the three intensity scaling approaches shows that the DICOM MU weighting (A) produces the most consistent relative peak intensities, as one would expect, while both CMOS (B) and QuARC (C) intensity measurements show a greater variability between the measured intensity and that needed for a smooth SOBP plateau, with the CMOS sensor weighting method exhibiting the most pronounced irregularities due to detector saturation effects at higher energies. **Figures 9D, E** show SOBP profiles for energy measurements made for the energy layers of the 5×5 spot box field weighted by the DICOM MU weights and QuARC intensity, respectively. Once again, the DICOM MU-weighted SOBP results in a flatter plateau, with some inhomogeneities in the plateau due to uncertainties in the range measurements. By comparison, the QuARC intensity weighted SOBP produces a much weaker top energy layer, and hence fails to properly reconstruct the SOBP plateau and distal edge.

### 3.4 Monte Carlo dose reconstruction

#### 3.4.1 Single spot position SOBP

The first PBS field to be reconstructed using TOPAS was the single spot position SOBP consisting of 34 different pencil beams, each with a different energy but delivered at the same position. The purpose of the single spot position reconstruction was to evaluate the accuracy of the longitudinal profile reconstruction, as well as the single spot position lateral profiles, as measured by the QuADProBe. However, as mentioned previously, the delayed start of the QuARC acquisition for the SOBP plan delivery meant that the distal energy layer of 159.55 MeV was not recorded, resulting in a substantial distortion to the planned SOBP dose due to the significant fraction of the overall dose that is delivered in the distal layer. As such, the comparison between the QuADProBe and DICOM reconstructed dose distributions was performed both with the full DICOM parameters of the 34 pencil beams, as well as for the 33 matched DICOM energies, excluding the distal energy layer.

**Figure 10:**
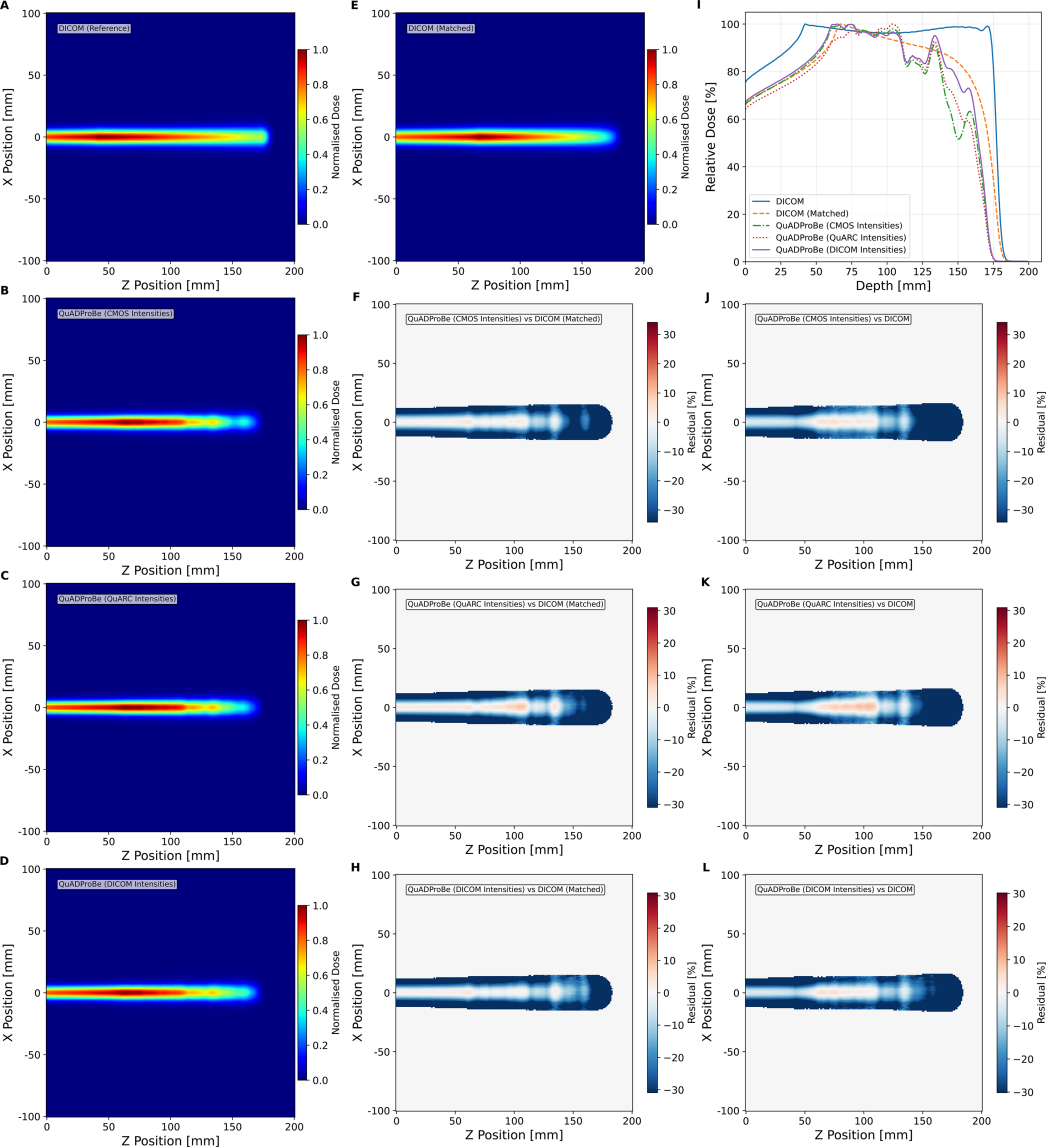
Longitudinal normalised dose profiles for the single-spot SOBP reconstruction. The 2D longitudinal normalised dose profile in the ZX plane (y-slice at 0,mm) was generated using **(A)** complete DICOM delivery parameters and QuADProBe input parameters with intensity scaling from **(B)** CMOS, **(C)** QuARC, and **(D)** DICOM values. **(E)** shows reconstruction using DICOM beam parameters adjusted to match the number of energy layers detected by QuARC. **(F–H)** display the corresponding residuals between this distribution and those derived from measured input parameters. **(I)** presents the combined PDD from MC simulation using QuADProBe-measured values (CMOS pixel sensor, QuARC intensity, and DICOM MU weights for intensity scaling) compared with DICOM input parameters. **(J–L)** show the respective residuals between the complete DICOM ZX distribution and distributions from measured input parameters.

**Figure 10I** shows the integrated percentage depth dose (PDD) curve, generated by summing the individual Bragg Peak PDDs and weighting each individual spot by either the DICOM MU values, the amplitude of the Gaussian fit applied to the lateral beam profile from the CMOS pixel sensor or the integrated charge intensity of all of the photodiodes from the QuARC detector. **Figure 10** also shows corresponding 2D depth dose profiles in the ZX-plane, using the QuADProBe input parameters (also scaled by the corresponding weightings) and their residuals compared to both the full DICOM input parameters, and the matched DICOM input parameters which omit the distal energy layer. The resulting *R*_80_ values for each of the simulations are shown in **Table 2**: the QuADProBe reconstructed SOBP has a significantly shorter range than that from the planned SOBP due to the distal layer. A considerably better match is seen in **Figure 10I** when the distal layer is omitted from the reconstructed DICOM energies.

**Table 2:** Table showing the longitudinal beam parameters — proton range (*R*_80_), SOBP width and distal dose fall off from the dose distributions in **Figure 10** — and the lateral beam profile parameters: the beam spot position and size obtained from the *µ* and *σ* of the Gaussian fit, respectively, at 10 mm, 100 mm and 150 mm depths from the TOPAS dose reconstruction of the single spot position SOBP field.

Parameter		QuADProBe intensities			DICOM	
		CMOS	QuARC	DICOM	Full	Matched
*R*_80_ (mm)		138.14 ± 0.56	139.16 ± 0.28	146.80 ± 0.81	175.16 ± 0.08	155.76 ± 1.14
SOBP width (mm)		55.72 ± 1.08	52.25 ± 0.85	56.71 ± 1.24	142.09 ± 1.54	69.65 ± 3.39
Distal dose falloff (mm)		31.69 ± 0.56	30.04 ± 0.28	23.45 ± 0.81	4.49 ± 0.08	21.62 ± 1.14
*µ_x_* (mm)	10 mm depth	-0.476 ± 0.006	-0.460 ± 0.006	-0.460 ± 0.006	-0.522 ± 0.007	-0.521 ± 0.007
	100 mm depth	-0.439 ± 0.007	-0.429 ± 0.007	-0.422 ± 0.007	-0.501 ± 0.007	-0.506 ± 0.007
	150 mm depth	-0.381 ± 0.007	-0.346 ± 0.007	-0.351 ± 0.007	-0.519 ± 0.007	-0.510 ± 0.007
*µ_y_* (mm)	10 mm depth	-0.399 ± 0.007	-0.420 ± 0.007	-0.421 ± 0.007	-0.512 ± 0.007	-0.511 ± 0.007
	100 mm depth	-0.387 ± 0.007	-0.416 ± 0.007	-0.405 ± 0.007	-0.501 ± 0.007	-0.503 ± 0.007
	150 mm depth	-0.315 ± 0.007	-0.436 ± 0.007	-0.422 ± 0.007	-0.509 ± 0.007	-0.510 ± 0.007
*σ_x_* (mm)	10 mm depth	3.498 ± 0.005	3.479 ± 0.005	3.490 ± 0.005	3.930 ± 0.005	3.913 ± 0.005
	100 mm depth	4.007 ± 0.005	4.013 ± 0.005	3.976 ± 0.005	4.234 ± 0.006	4.341 ± 0.006
	150 mm depth	4.695 ± 0.006	4.748 ± 0.006	4.737 ± 0.006	4.899 ± 0.006	4.959 ± 0.006
*σ_y_* (mm)	10 mm depth	3.882 ± 0.005	3.843 ± 0.005	3.850 ± 0.005	3.890 ± 0.005	3.885 ± 0.005
	100 mm depth	4.331 ± 0.006	4.319 ± 0.006	4.281 ± 0.006	4.295 ± 0.006	4.362 ± 0.006
	150 mm depth	4.961 ± 0.006	4.975 ± 0.006	4.972 ± 0.006	5.012 ± 0.006	5.067 ± 0.006

The resulting underestimate in the QuADProBe range of 8.96–17.62 mm is significantly worse than that shown in **Figure 8**. However, this can clearly be attributed to the distortion of the SOBP plateau due to the missing distal energy layer leading to a large discrepancy in the resulting *R*_80_. In addition to a shorter range, the QuADProBe reconstruction also resulted in a narrower SOBP width compared to the DICOM reconstruction, again due to the omission of the highest energy pencil beam and the differences in the intensity of the distal edge Bragg peaks. Furthermore, it can be seen that the flatness of the SOBP plateau is noticeably less smooth for the QuADProbe reconstructed profiles compared to that of both the full and matched DICOM PDD curves in **Figure 10I**, owing to both inaccuracies in the reconstructed QuARC depth dose curves and the impact of the CMOS sensor saturation on the spot weight calculation, as shown in **Figure 9**.

**Figure 11:**
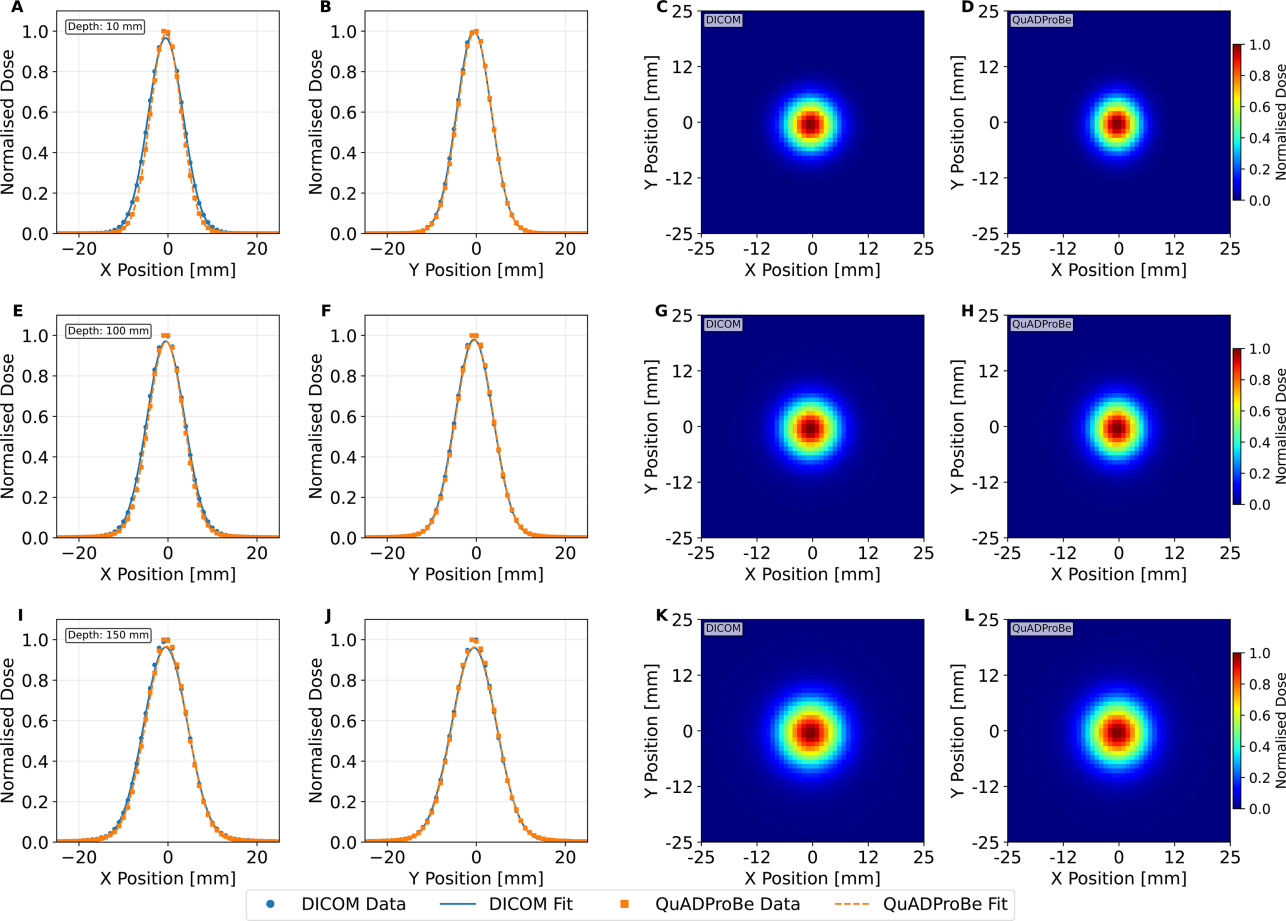
Lateral 1D horizontal **(A, E, I)** and vertical **(B, D, J)** normalised dose profiles taken across the centre of the dose distribution with a Gaussian fit applied, and the corresponding 2D normalised dose profiles for both the DICOM parameters **(C, G, K)** and QuADProBe parameters **(D, H, L)** at depths of 10 mm **(A–D)**, 100 mm **(E–H)** and 150 mm (*R*_80_) **(I–L)** from the TOPAS 3D dose reconstruction of the single spot position SOBP.

**Figure 11** shows the lateral profiles for both 1D horizontal and vertical slices across the centre of the beam, along with the corresponding 2D profiles at depths of 10 mm (entrance region), 100 mm (mid-SOBP plateau) and 150 mm. The corresponding values of the beam position and size, obtained from the mean ( $\mu_{x,y}$) and standard deviation ( $\sigma_{x,y}$) of the Gaussian fit applied to the 1D profiles, along with associated uncertainties on these values from the fit itself, are shown in **Table 2**. An excellent agreement can be seen between QuADProBe and DICOM measurements for beam size parameters, with $\sigma_{x}$ and $\sigma_{y}$ showing average agreements of *>*92% and *>*98% respectively across all depths and weighting schemes. The use of the QuARC intensities for scaling the individual Bragg peak weights demonstrated the best overall performance with 89.1% agreement across all lateral profile parameters when compared to DICOM Full data. The beam sizes obtained from the QuADProBe reconstruction showed consistency with those from the DICOM parameter reconstruction, with the smallest discrepancies observed for *σ_y_* at all depths (*<* 2% difference). For *σ_x_*, the largest disagreement was 11.5% at 10 mm depth, reducing to 5.4% at 100 mm and 4.2% at 150 mm, indicating improved agreement with increasing depth for horizontal beam sizes.

The beam position parameters showed greater variability, with $\mu_{y}$ exhibiting the largest discrepancies, particularly for CMOS weighting (up to 38% at 150 mm depth). The $\mu_{x}$ measurements showed better agreement, with discrepancies ranging from 8.6-15.8% depending on depth and weighting scheme. Overall, position accuracy decreased with increasing depth. This agrees with the fact that the CMOS sensor measured beam sizes were non-symmetrical upstream from the isocentre, as discussed earlier and shown in **Supplementary Figures S2**, **S4**.

#### 3.4.2 Box field

**Figure 12:**
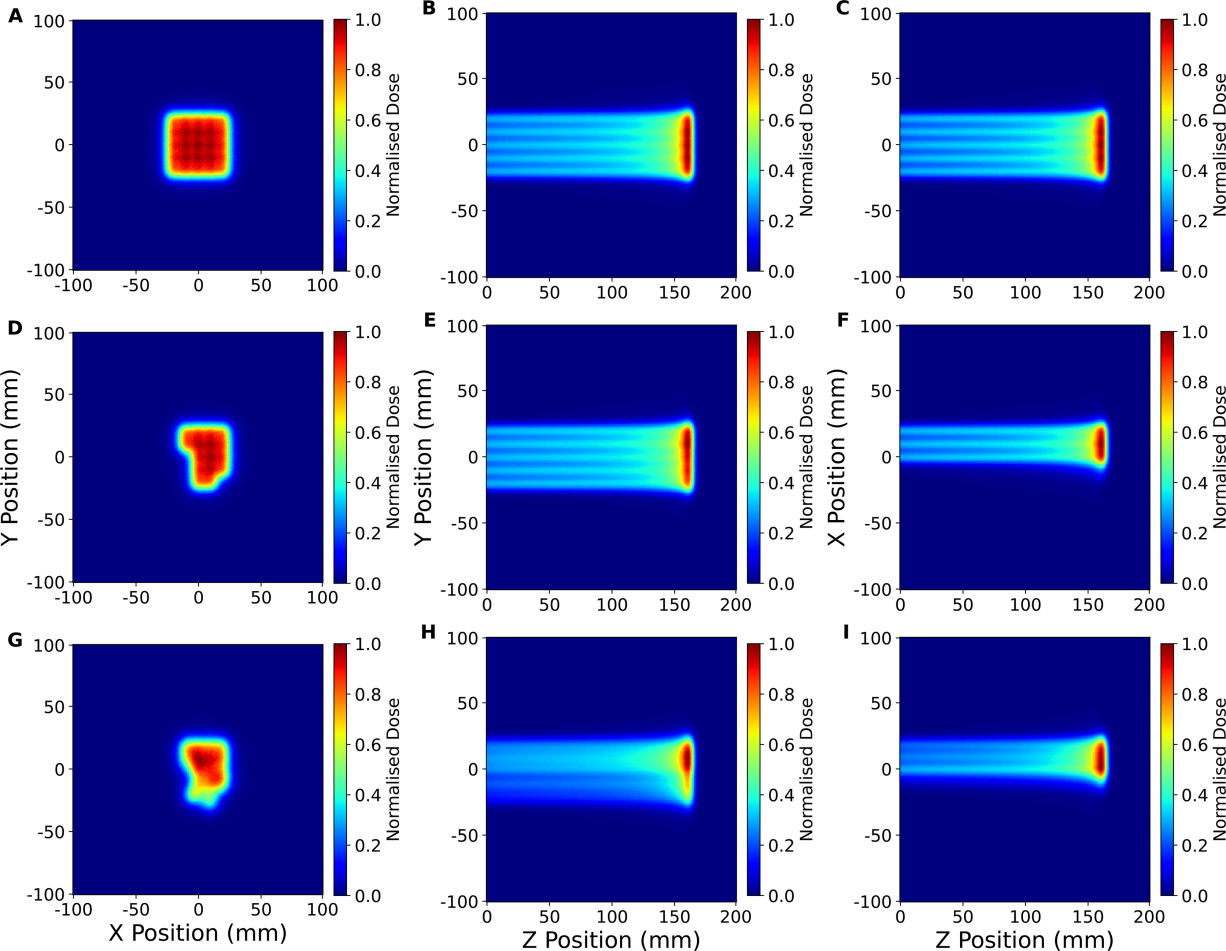
2D profiles of full box field 3D reconstruction using the full DICOM parameters **(A–C)**, partial DICOM parameters corresponding to the spots measured by the QuADProBe **(D–F)** and the QuADProBe measurements **(G–I)** demonstrating the spots missed or unable to be analysed by the CMOS.

The box field delivered to the detector consisted of a 5×5 spot pattern with lateral spot positions between −2 cm and +2 cm, with 1cm spacing in both the horizontal and vertical direction, as shown in **Figures 12A–C**. An identical spot pattern was delivered to each of 10 energy layers ranging from 126.26 MeV to 152.78 MeV. However, due to the frame rate of the CMOS pixel sensor, as well as damaged pixels on the detector, only the spots in the highest energy layer (152.78 MeV) could be reconstructed. Furthermore, within this energy layer only 16 out of the 25 delivered spots, shown in **Figures 12D–F**, were measured successfully by the CMOS detector, as can be seen in **Figures 12G–I**, with some spots being measured between positions. As such, to enable a more useful comparison of the planned and reconstructed lateral profiles and assess the potential reconstruction performance of the QuADProBe, only the top-left 3×3 spots were selected for comparison.

**Figure 13:**
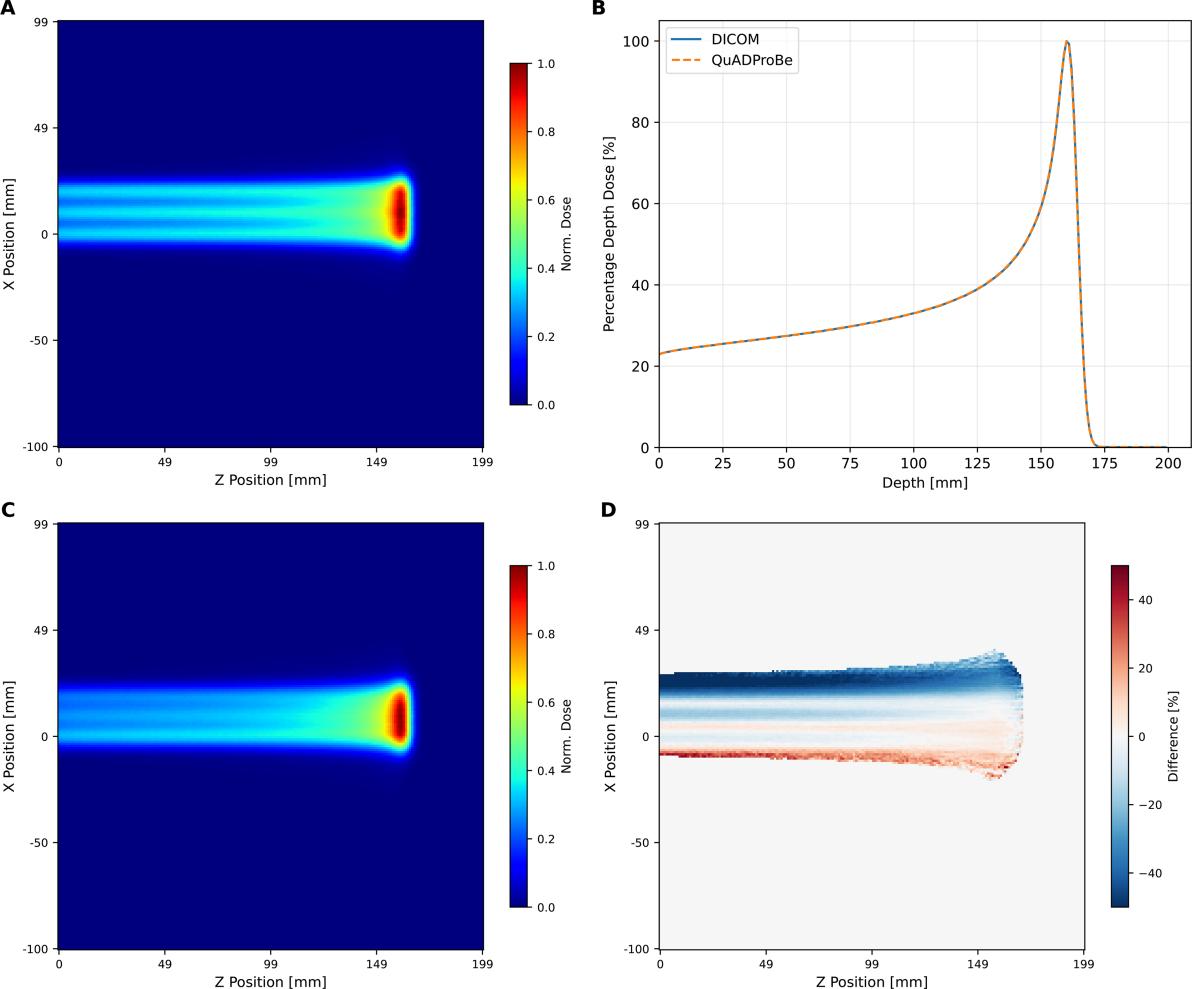
Longitudinal normalised dose profiles from TOPAS 3D dose reconstruction of the box field. **(A)** The 2D normalised longitudinal profile along the ZX plane taken from a y-slice at 0 mm using the DICOM input parameters. **(B)** Plot of combined PDD from the MC simulation using the QuADProBe measured values and DICOM values as input parameters. **(C)** The 2D normalised longitudinal profile along the ZX plane taken from a y-slice at 0 mm using the QuADProBe input parameters; the corresponding residuals between **(C, A)** are shown in **(D)**.

**Figure 13** shows the integrated percentage depth dose (PDD) curve, and corresponding 2D profiles in the ZX-plane, using the DICOM and QuADProBe input parameters. The *R*_80_ values for the QuADProBe and DICOM reconstructed distributions are in extremely close agreement, with a difference of 0.1 mm; the distal dose fall off values are also within their respective uncertainty ranges.

**Figure 14:**
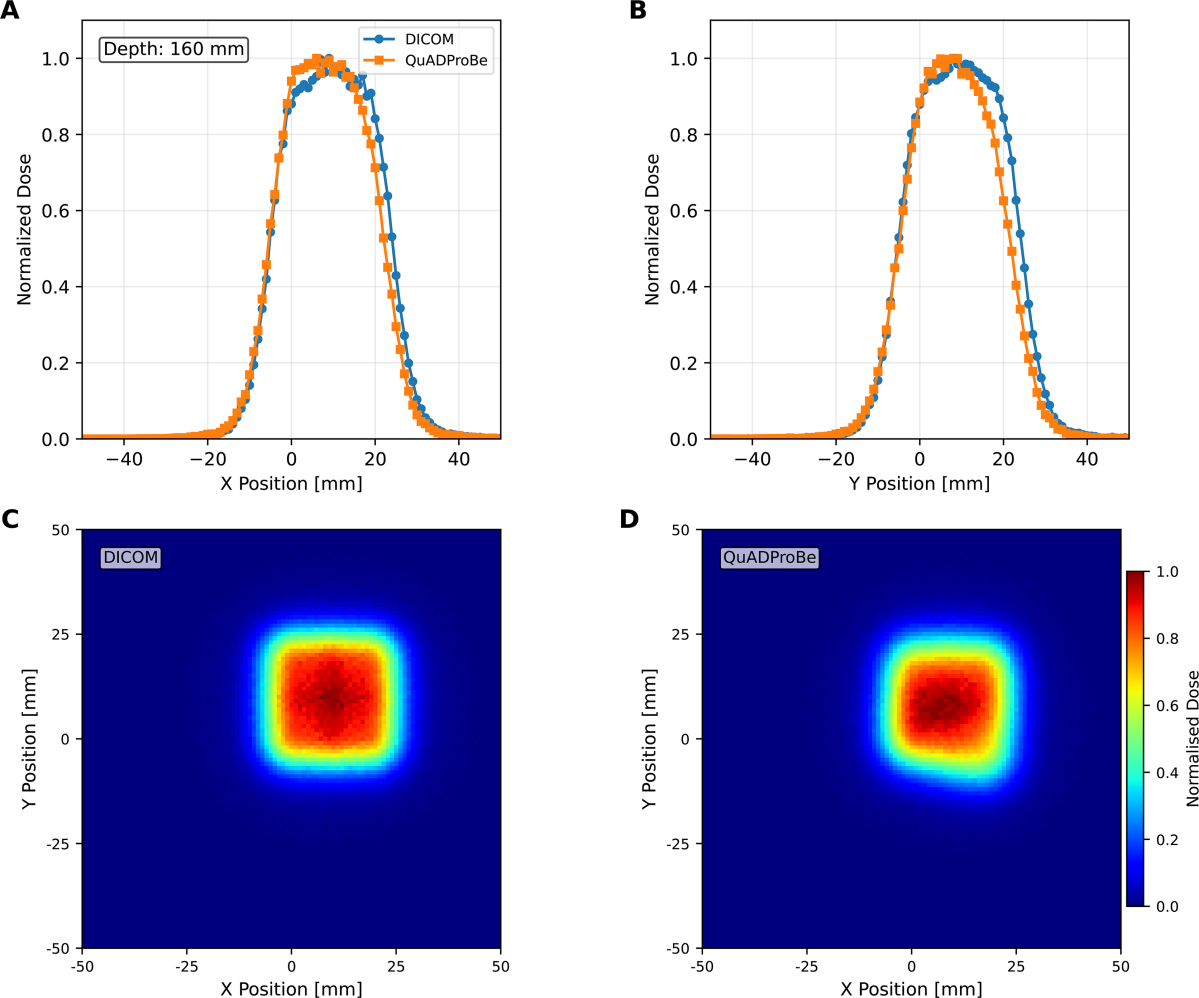
Lateral 1D horizontal **(A)** and vertical **(B)** normalised dose profiles taken across the centre of the dose distribution, and the corresponding 2D normalised dose profiles for both the DICOM parameters **(C)** and QuADProBe parameters **(D)** at a depth of 150 mm from the TOPAS 3D dose reconstruction of the box field.

For the box field, the lateral profiles were only analysed and compared at *R*_80_ depth, since this dose reconstruction only consisted of one energy layer, for reasons previously mentioned. The 1D horizontal and vertical lateral profiles, along with the corresponding 2D profiles for the QuADProBe and DICOM dose reconstruction are shown in **Figure 14**. The comparative metrics for the box field were the full width at half maximum (FWHM), the penumbra width (between 80% and 20%), and the flatness and symmetry across the box field. These parameters for the box field lateral profiles are shown in **Table 3**. There are larger differences between the QuADProBe and DICOM reconstructed lateral profiles, particularly with the FWHM being smaller for the QuADProBe profile, and all penumbra widths being larger for the QuADProBe reconstruction. Despite these discrepancies, the flatness was the same within errors for both horizontal and vertical.

**Table 3:** Table showing the longitudinal and the lateral beam profile parameters: full-width half maximum (FWHM), the penumbra width (80% to 20%), the flatness and the symmetry across the box field (at 80% of FWHM), at 160 mm (*R*_80_) depth from the TOPAS dose reconstruction of the box field.

Parameter	QuADProBe	DICOM
*R*_80_ (mm)	162.95 ± 0.05	163.05 ± 0.06
Distal Dose Falloff (mm)	3.83 ± 0.06	3.78 ± 0.06
FWHM*_x_* (mm)	28.13 ± 0.08	29.66 ± 0.06
FWHM*_y_* (mm)	27.10 ± 0.10	29.86 ± 0.09
Left Penumbra Width (mm)	7.52 ± 0.11	7.22 ± 0.09
Right Penumbra Width (mm)	8.37 ± 0.23	7.77 ± 0.17
Top Penumbra Width (mm)	8.06 ± 0.14	7.23 ± 0.12
Bottom Penumbra Width (mm)	8.76 ± 0.16	7.57 ± 0.16
Flatness*_x_* (%)	11.27 ± 0.70	11.43 ± 0.70
Flatness*_y_* (%)	11.65 ± 0.69	11.39 ± 0.70
Symmetry*_x_* (%)	0.73 ± 0.10	1.55 ± 0.09
Symmetry*_y_* (%)	0.56 ± 0.10	1.92 ± 0.09

**Figure 15:**
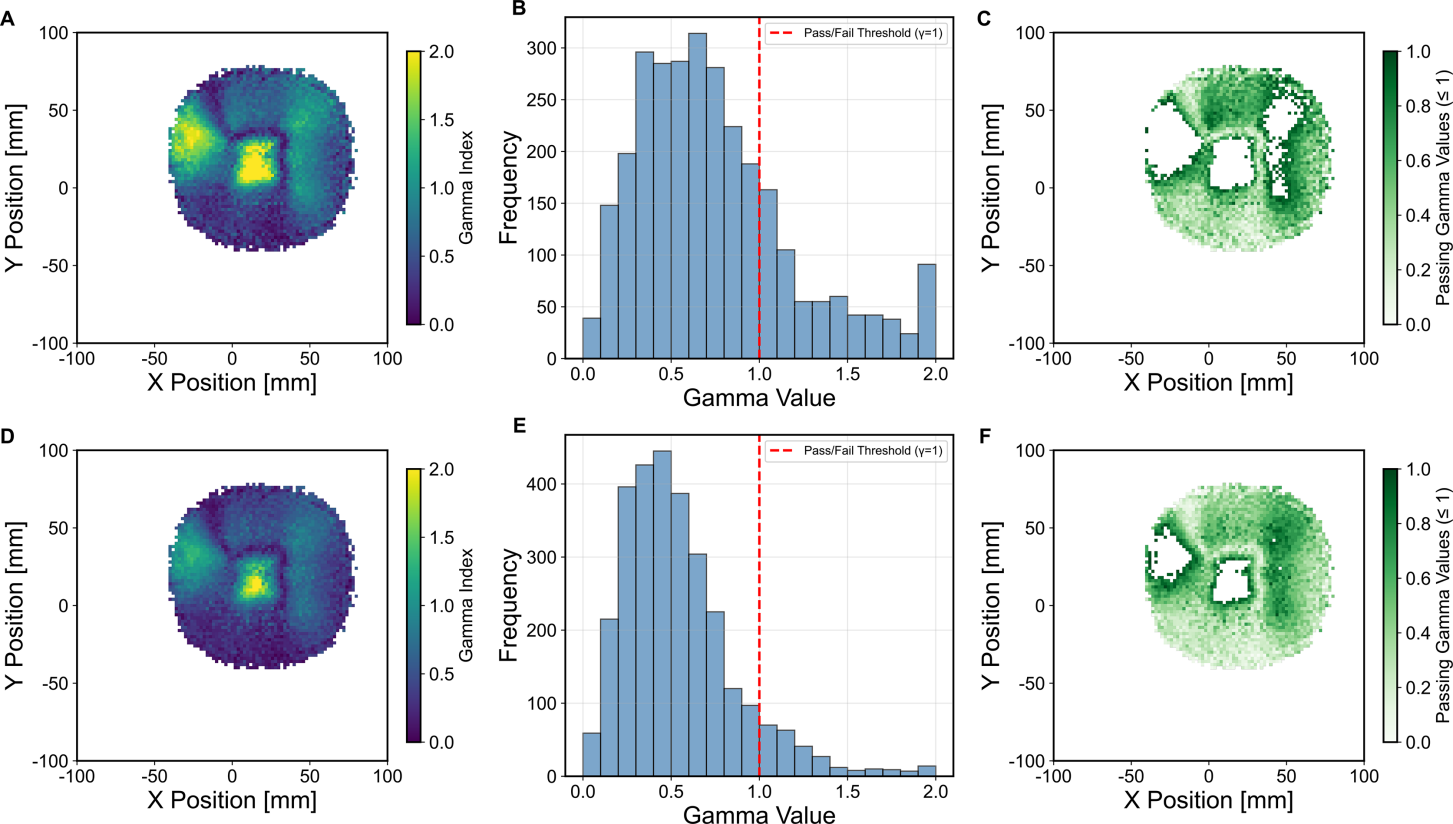
Gamma analysis comparing the TOPAS simulated 2D lateral dose profiles from the QuADProBe and DICOM parameters for the box field at *R*_80_ using a 2%/2mm and 3%/3mm criteria with a 5% dose cut-off threshold. **(A, D)** Gamma index map showing the distribution of gamma values across the field, where values ≤ 1 indicate passing points for 2%/2mm and 3%/3mm, respectively. **(B, E)** Histogram of gamma values showing the frequency distribution, with the pass/fail threshold (*γ* = 1) indicated by the red dashed line for 2%/2mm and 3%/3mm, respectively. **(C, F)** Pass/fail map highlighting regions that pass the gamma criteria (green areas) for 2%/2mm and 3%/3mm, respectively.

Additionally, a gamma index analysis was performed on the box field lateral profile in order to evaluate the uniformity of the broad field between the QuADProBe and DICOM reconstructions. A 2%/2 mm criteria was used for the analysis based on the proton therapy PBS QA recommendations for the evaluation of broad beam uniformity (14), and a 5% dose threshold was used to allow optimal evaluation of field edge matching while minimising the impact of the MC simulation statistical noise. The gamma passing rate (i.e. *γ* ≤ 1) was determined to be 77.00%. Using a more lenient criteria of 3%/3mm give a pass rate of 91.11%. The 2%/2 mm passing rate is below the clinically acceptable pass rate (14), because of the discrepancies between the measured and prescribed beam sizes and positions. The distributions of the gamma index values are shown in **Figure 15**, where it can be seen that the largest disagreement between the profiles was across near the centre of the box field, due to the smearing of the QuADProbe reconstructed profile from inaccuracies in the size and position determination of the pencil beam spots by the CMOS pixel sensor.

## 4 Discussion

The work in this paper presents proof-of principle measurements for a phantom-less approach to proton PBS PSQA that utilises a novel integrated detector, the QuADProBe, intended to provide spot-by-spot measurements of a full PBS treatment plan. Proof-of-principle experimental measurements were performed using a Varian ProBeam clinical proton beam at the UCLH Proton Therapy Centre. The measurements involved the use of standardised QA fields, namely a single spot position SOBP and a 5×5 spot box field, for the purpose of validating the approach using recommended PBS QA comparative metrics (14) and highlighting areas that need improvement for the continued development of both the detector and this approach.

The response of the TC successfully demonstrated excellent linearity with the prescribed dose ( $R^{2}=1.000$) for a 3×3×3 spot cube as shown in **Figure 6C**, showing that it is capable of providing reliable integrated dose measurements for the delivered beam fields. Although it should be noted that this is limited to three data points or 2, 5, and 10 Gy. While full linearity of the calorimeter is assumed, further tests are required. While the current timing resolution does not allow for individual spot dose verification, the TC serves as an important component for overall field dose validation and provides independent verification of the total delivered dose. This integrated dose measurement capability complements the spot-by-spot parameter verification from the QuARC and CMOS detectors, contributing to the comprehensive quality assurance approach of the QuADProBe system.

The beam spot size and position measurements made with CMOS pixel sensor detector demonstrated the ability to provide measurement of each spot for the single spot position SOBP when utilising a 120 mm by 20 mm ROI, meaning a 230 Hz acquisition could be used. The single-spot position SOBP beam size measurements, shown in **Figure 7**, are smaller than those back-projected from the DICOM file, in addition to showing significant fluctuation in the beam size and beam spot position. However, for higher energy spots in the SOBP field, the CMOS detector experienced saturation, as shown in **Figure 7A** for the 147.52 MeV spot, though Gaussian fitting remained possible to extract beam parameters.

Some of the variations between the measured and prescribed spot size can also be attributed to the fact that the DICOM files were generated for gantry 1 at UCLH, and the majority of the full detector measurements were performed in gantry 3. This was caused by the couch position interlock on gantry 1 during the second evening of measurements, as mentioned in Section 2.5. **Supplementary Figure S4** shows a comparison of the beam spot shape measured using an XRV-3000 during beam QA from gantry 1 and 3. A comparison between the DICOM file proton beam spot sizes for a range of energies with those measured using an XRV-3000 during beam QA at a gantry angle of 270° at both gantries is shown in **Supplementary Figure S5**: note the visible deviation for gantry 3. Subsequently, the CMOS detector experienced saturation, as shown in **Figure 7A** for the 147.52 MeV spot. However, it was still possible to apply a Gaussian fit to the saturate profiles in order to extract beam parameters. For the 5×5 box field measurements, the detector’s limitations became more apparent. Due to the larger field requiring full frame readout at 35 fps and pixel damage issues, only 16 out of 25 spots were successfully measured, necessitating analysis of a reduced 3×3 spot box field configuration.

The range measurements that were made with the QuARC scintillator-based range telescope and the corresponding reconstructed Bragg curves showed a good agreement with the reference Bragg curves from UCLH PBT centre reference curves. Additionally, the beam energies obtained from these range measurements demonstrated a strong correlation with the DICOM energy values (*R*^2^ *>* 0.998). However, there was an offset of −1.89 mm and −7.83 mm for the single spot position SOBP field and box field, respectively. These offsets can be attributed to both the uncertainty in the individual thicknesses of the scintillator sheets within the QuARC as well as the accuracy of the WET calculation for the detector components in front of the QuARC detector, particularly for the box field which covered a larger transverse area and hence likely scanned over components on the TC where the WET was not as well characterised.

The dose reconstruction of the single spot position SOBP field, shown in **Figure 10**, using MC simulations with the QuADProBe measured parameters showed reasonable agreement with DICOM reference simulations for longitudinal profiles. The reconstructed SOBP demonstrated a noticeably shorter range than the full DICOM dose reconstruction shown in **Figure 10I**. The primary cause of this discrepancy was the failure to acquire the highest energy (159.55 MeV) in the planned delivery as discussed previously. This compromised the SOBP reconstruction, creating a pronounced dose deficit in the proximal region and reducing the overall SOBP width. This is apparent in **Figure 9** that shows the individual Bragg curves and their weighted contributions to the SOBP across different intensity scaling methods. The visible differences in SOBP plateau homogeneity reflect the combined effects of the missing data, intensity scaling variations from detector saturation, and residual range measurement uncertainties. The relative intensities used for scaling the individual Bragg peaks demonstrate how different weighting schemes (DICOM, CMOS, QuARC) produce varying SOBP shapes, with CMOS intensity scaling showing the most pronounced irregularities due to saturation effects at higher energies.

When comparing the QuADProBe reconstructed dose distributions with the DICOM reconstruction that omitted the highest energy Bragg peak to match the acquired data, the range discrepancies became more apparent. The *R*_80_ values from the QuADProBe reconstruction were 8.96–17.62 mm shorter than the matched DICOM reconstruction (depending on intensity weighting scheme), substantially larger than the 2 MeV energy offset alone would suggest. Similarly, the SOBP width from the QuADProBe reconstruction was approximately 15 mm shorter than the matched DICOM reconstruction. These enhanced discrepancies arise from the combined absence of the highest intensity proximal Bragg peak and variations in the measured intensities of the remaining distal peaks, which together suppress the distal dose falloff and significantly impact the *R*_80_ determination. The lateral profiles at various depths, shown in **Figure 11**, for the single spot position SOBP field showed that despite differences in beam sizes between those measured by the CMOS pixel sensor and those from the DICOM, the MC reconstructed lateral profiles converge toward closer agreement at greater depths.

Although the dose reconstruction of the 5×5 box field had to be reduced to a single energy layer due to the limitations of the CMOS pixel sensor, the range of the reconstructed energy layer between the QuADProBe and DICOM simulations agreed within less than 0.1 mm. The broad field lateral profile reconstructions for the box field in **Figure 14** revealed greater challenges, where gamma analysis yielded a 77% pass rate using 2%/2mm criteria (improving to 91.11% at 3%/3mm). This relatively low pass rate can be attributed to the combination of measurement uncertainties in beam spot sizes and positions from the CMOS detector, along with the energy offset affecting the overall dose distribution shape.

The primary advantage of this proposed approach for PSQA is the ability to measure the necessary QA parameters (i.e. energy, spot size, spot position, and dose) within a single beam delivery. Furthermore, it is able to provide independent verification of beam delivery parameters rather than relying solely on machine log-files while providing simultaneous log-file verification without any increase in beam delivery time. Unlike traditional measurement-based PSQA that requires modified treatment plans and phantom setups, the QuADProBe approach will eventually be able to verify the full treatment delivered to the actual patient geometry through phantom-less dose reconstruction. Monte Carlo dose reconstruction represents the most computationally intensive step. However, by parallelising across available computing resources, the wall-clock time for complete field reconstruction remains comparable to single-spot simulation time rather than scaling linearly with spot count. For the field configurations tested, parallel processing yielded complete reconstructions within 1–4 hours. Future optimisation through GPU-accelerated MC simulation codes such as FRED, which is capable of simulating 10^6^ primaries per second on a single GPU card (56) could reduce reconstruction times to under 5 minutes for a full patient treatment plan (using 1% of the total protons used in the actual delivery).

This methodology offers significant potential time savings compared to conventional measurement-based approaches while providing enhanced verification capabilities compared to pure log-file methods. However, the current proof-of-concept system faces several technical challenges that must be addressed before clinical implementation.

The offset in the QuARC range measurements demonstrates the importance of requiring an improved accuracy in the measurement of the individual scintillator sheets to ensure uniform thickness and light output as well as a more through characterisation of the WET of the TC and CMOS. Furthermore, the noisy electronics in the QuARC photodiode readout also impacted the accuracy and consistency of the energy measurements. The CMOS pixel sensor detector saturation at some of the higher intensities and slow frame rate that lead to incomplete field coverage for broad field delivery patterns indicate the need for detector improvements to avoid the need for modified acquisition strategies. Critically, the manual triggering system resulted in the loss of the highest energy layer in the SOBP field, demonstrating that a synchronised, automated acquisition trigger system is essential for clinical implementation. Furthermore, for the single spot position SOBP field, the beam size measurements were smaller than those that were back-projected from the DICOM beam sizes at isocentre. It is important to note, however, that these shortcomings do not constitute a fundamental limitation on the measurement principle and are all addressable — and achievable — with improved detector performance.

Clinical translation of this approach will require validation with realistic treatment plans beyond the simple QA fields tested in this study. The current system demonstrated feasibility with basic field configurations, but clinical plans involve more complex energy modulation, irregular field shapes, and varying spot weights that may present additional challenges for accurate parameter measurement and dose reconstruction. Part of the detector development pathway is the adaptation of the detector enclosure to be nozzle-mountable to enable QA measurements of a full PBS fraction with multiple fields.

Future development of the QuADProBe detector will involve a more sophisticated back-end readout board for the QuARC photodiodes to reduce the electronic noise. This will also involve using photodiodes on both sides of the QuARC detector, rather than just one, to be able to better characterise the Bragg Peak when scanning the beam laterally. Additionally, an alternative detector for the beam spot size and position measurements is being developed utilising a scintillating fibre (SciFi) profile monitor, based on the system under development for the Heidelberg Ion Therapy centre (57). A combined DAQ system is also under development to integrate the simultaneous acquisition of measurements from the QuARC, TC and SciFi profile monitor.

## 5 Conclusion

This proof-of-concept study demonstrates the feasibility of phantom-less patient-specific quality assurance for pencil beam scanning proton therapy using the QuADProBe integrated detector system. The system provided simultaneous spot-by-spot measurements of energy (*R*^2^ *>* 0.998 correlation with reference values), beam position, spot size, and intensity within a single beam delivery. Monte Carlo dose reconstruction using independently measured beam parameters showed good agreement with reference simulations for longitudinal profiles, validating the fundamental approach.

However, clinical implementation requires resolution of identified limitations including energy calibration offsets, CMOS detector saturation at higher intensities, acquisition synchronisation challenges, and lateral profile reconstruction accuracy (77% gamma pass rate at 2%/2mm for the box field). Ongoing detector development, including improved photodiode readout electronics, scintillating fibre profile monitors, and integrated data acquisition systems, will address these challenges. Once refined, this approach offers significant advantages over current PSQA methods by enabling independent verification of beam delivery parameters while maintaining patient geometry and reducing clinical resource requirements.

## Data Availability

The raw data supporting the conclusions of this article will be made available by the authors, without undue reservation.

## Footnotes

1 Whilst larger cases are available, the Peli 1510 has the advantage of fitting within the majority of airline carry-on hand luggage restrictions, making transportation for beam tests significantly easier.
