# Correction: A novel approach for proton therapy pencil beam scanning patient specific quality assurance using an integrated detector system and 3D dose reconstruction

**DOI:** 10.3389/fonc.2026.1834552

**Published:** 2026-05-01

**Authors:** Joseph J. Bateman, Sonia Escribano-Rodriguez, Samuel Flynn, Tony Price, Raffaella Radogna, Saad Shaikh, Harry Barnett, Connor Godden, Matthew Warren, Febian, Catherine Burne, Alison Warry, Lee Harrison-Carey, Colin Baker, Simon Jolly

**Affiliations:** 1 Department of Physics and Astronomy, University College London, London, United Kingdom; 2 Radiotherapy and Radiation Dosimetry Group, National Physical Laboratory, Teddington, United Kingdom; 3 School of Physics and Astronomy, University of Birmingham, Birmingham, United Kingdom; 4 Department of Physics, University of Bari, Bari, Italy; 5 Istituto Nazionale di Fisica Nucleare, Sezione di Bari, Bari, Italy; 6 Department of Medical Physics and Biomedical Engineering, University College London, London, United Kingdom; 7 Proton Beam Therapy Physics, University College London Hospital NHS Foundation Trust, London, United Kingdom

**Keywords:** proton therapy, pencil beam scanning, patient-specific quality assurance, integrated detector system, 3D dose reconstruction, Pencil Beam Scanning (PBS) PSQA, scintillator range telescope, CMOS pixel sensor

Affiliation “Istituto Nazionale di Fisica Nucleare, Sezione di Bari, Bari, Italy” was omitted for author Raffaella Radogna. This affiliation has now been added for author Raffaella Radogna.

The original version of this article has been updated.

