# Supplementary material for: A novel approach for proton therapy pencil beam scanning patient specific quality assurance using an integrated detector system and 3D dose reconstruction
Source: Front Oncol. 2025 Dec 8;15:1677439. doi: 10.3389/fonc.2025.1677439 (PMC12719259; doi:10.3389/fonc.2025.1677439)
Supplement: Supplementary file 1 [file DataSheet1.pdf]

Supplementary Material

1 SUPPLEMENTARY TABLES AND FIGURES

1.1 Figures

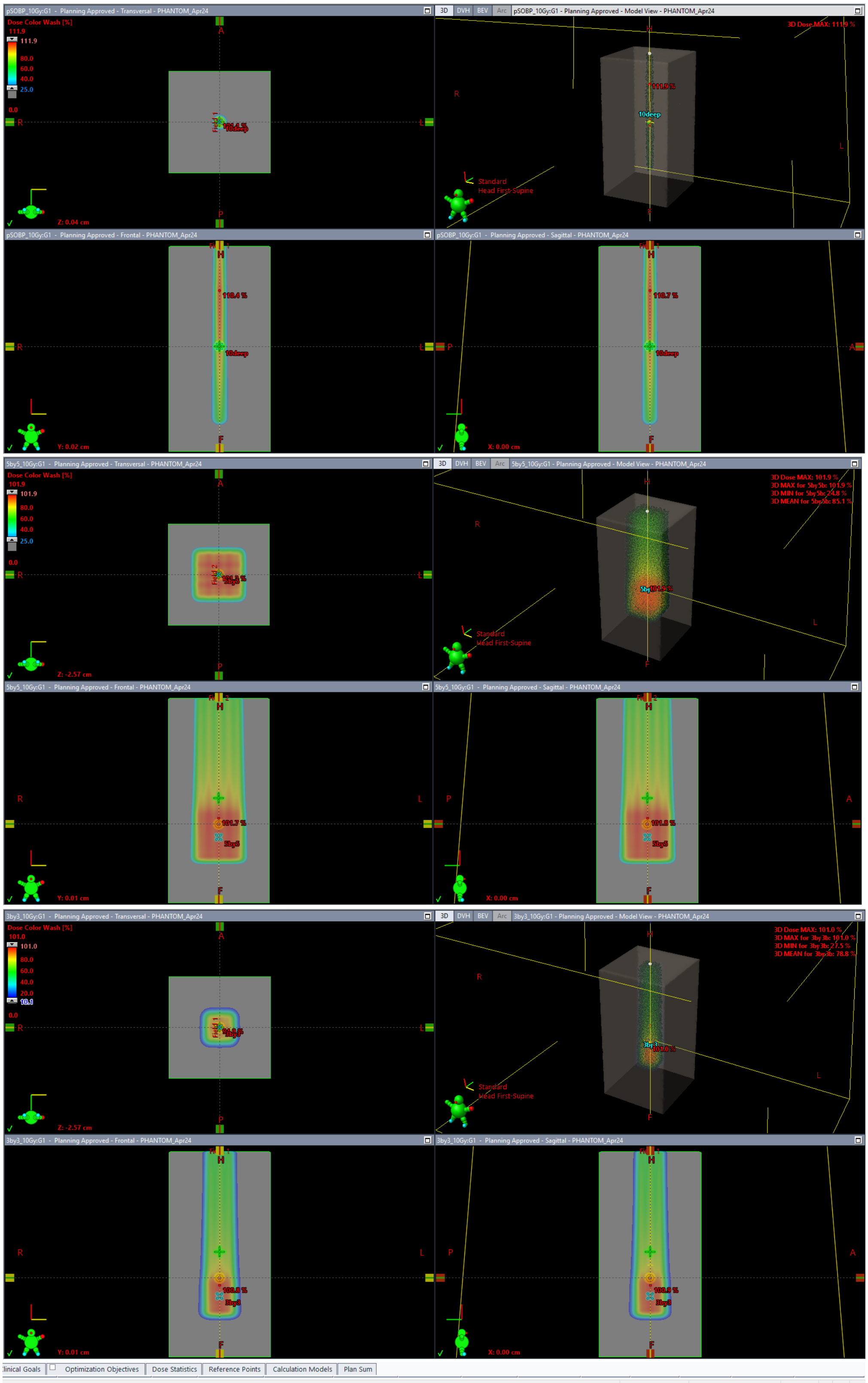

Figure S1. Images from the Varian Eclipse Treatment Planning System showing the prescribed QA plans that were used in this experiment. A 10 Gy single spot position SOBP (Top), a 10 Gy 5×5 spot box field (middle) and a 10 Gy 3×3 spot box field (bottom).

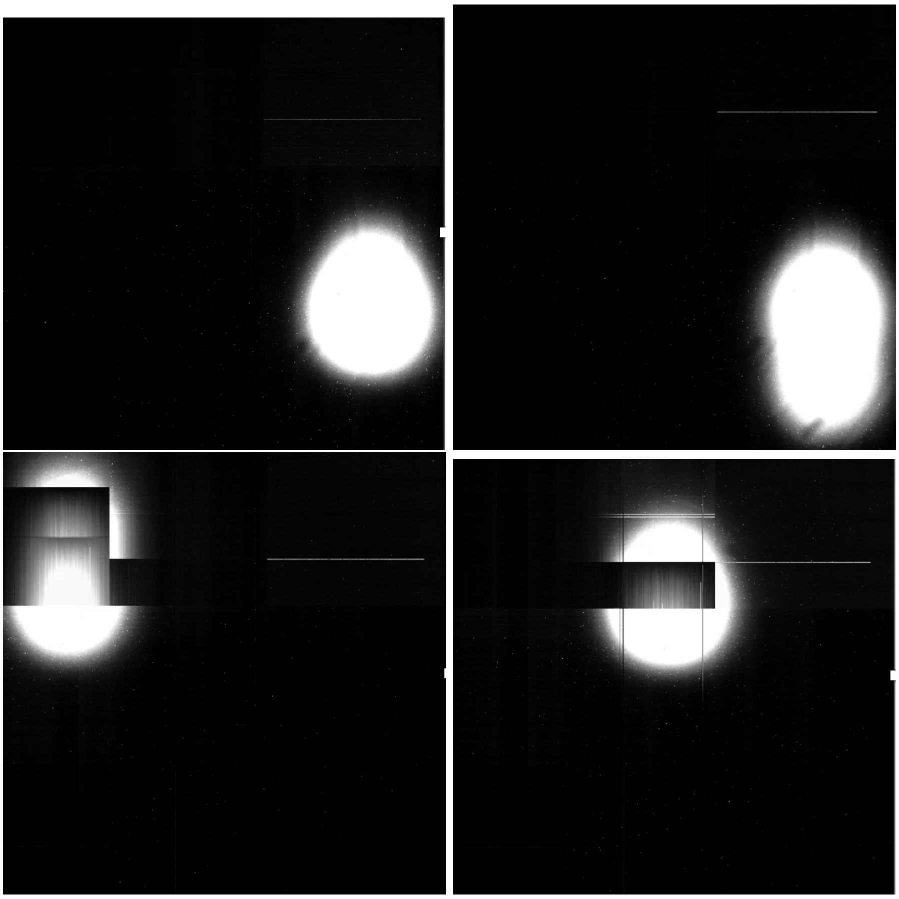

Figure S2. CMOS measured spots for 5×5 box field, showing (clockwise from top left) a standard spot, a distorted spot, damaged pixels and distorted spot over damaged pixels.

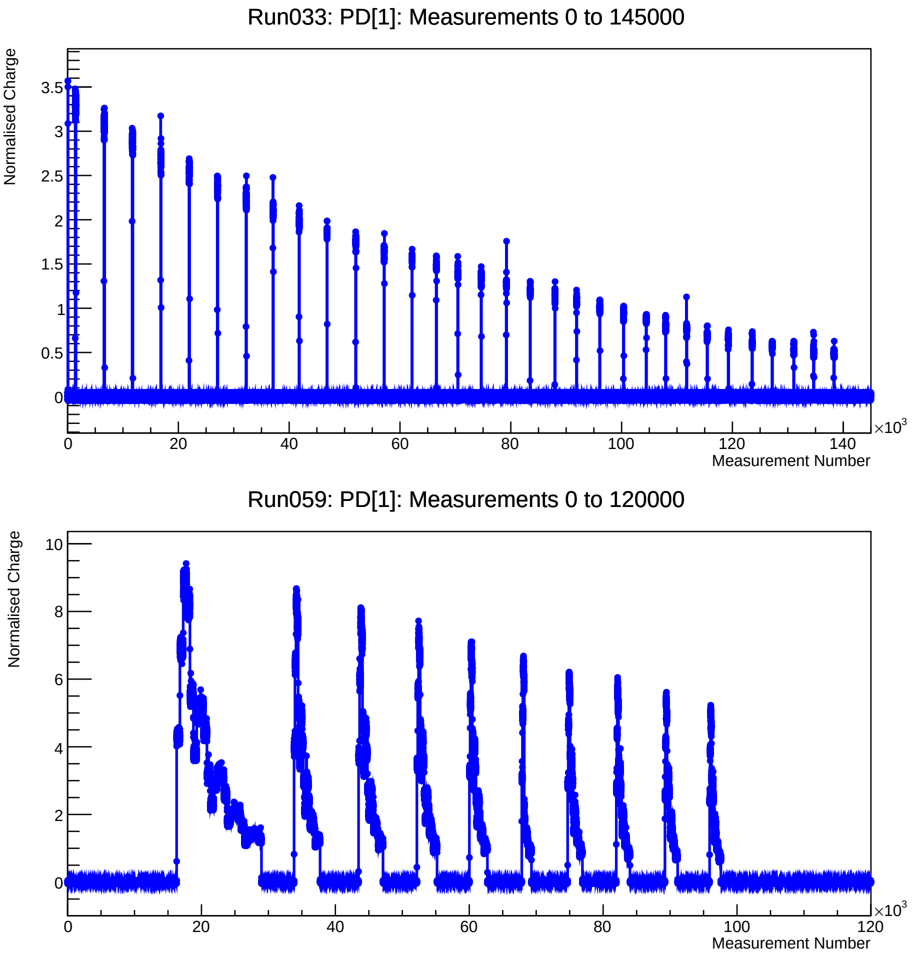

Figure S3. Plot showing the normalised charge readings for each measurement number (with each measurement lasting 170 μs), demonstrating the number of energy layers acquired by the QuARC range telescope. The top figure is for the single spot position SOBP field, where only 33 peaks are observed instead of the planned 34, with the first peak being very close to the acquisition start time. The bottom row is for the 5$\times$5$\times$10 box field with 10 energy layers.

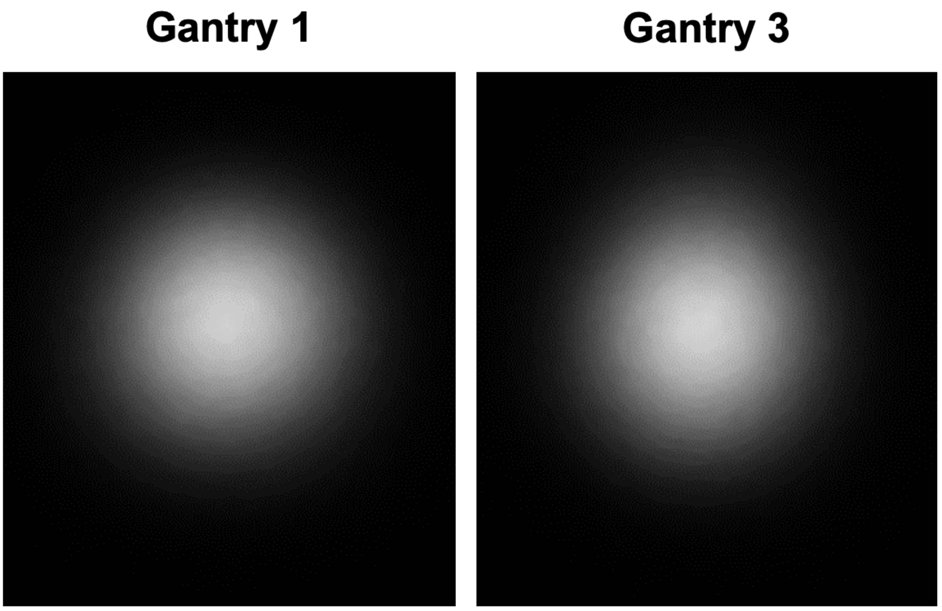

Figure S4. UCLH 2D beam profiles measured at the isocentre using an XRV-3000 detector for Gantry 1 and 3.

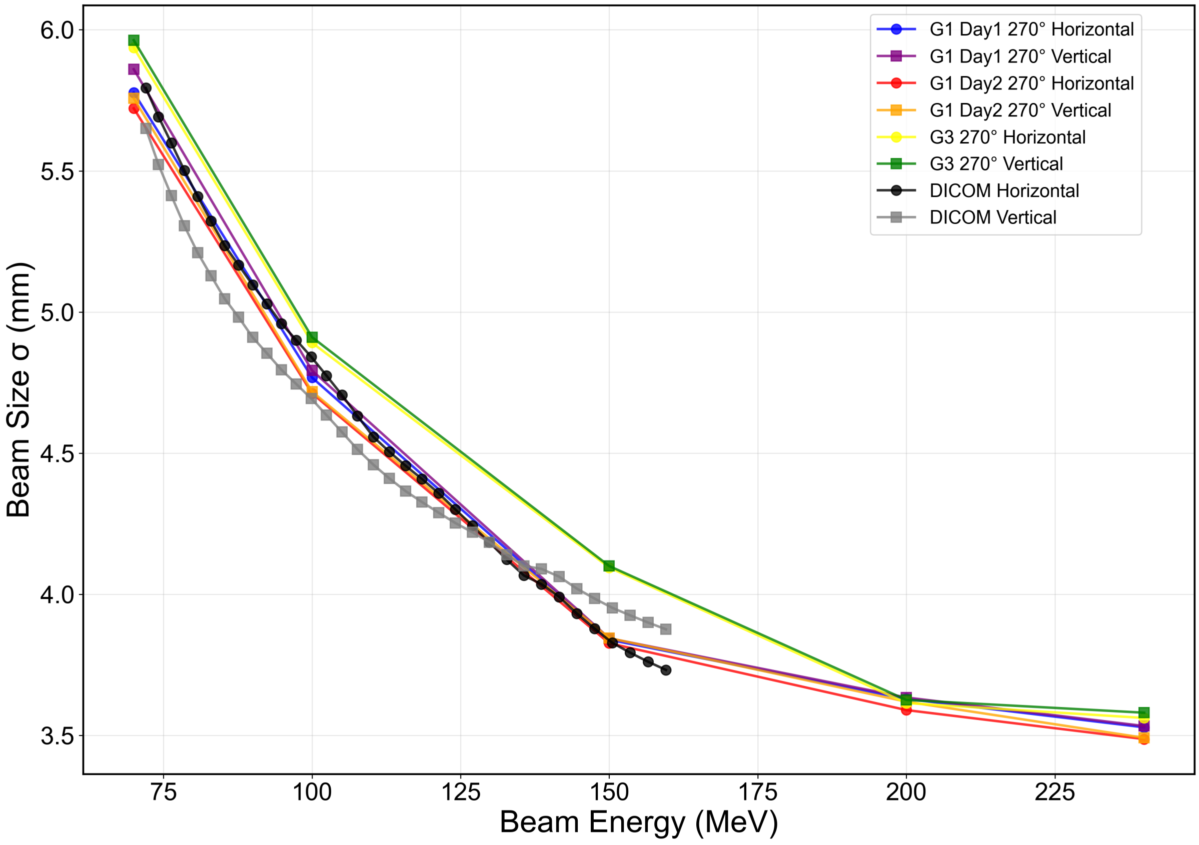

Figure S5. Plot showing a comparison of the proton beam spot size for each energy at UCLH between the DICOM prescribed values and the QA measured values using an XRV-3000 detector at a gantry angle of 270 degrees.
